# Mobile Intervention for Increasing COVID-19 Testing in K-12 Schools Serving Disadvantaged Communities: Randomized Controlled Trial of SCALE-UP Counts

**DOI:** 10.2196/79775

**Published:** 2025-11-11

**Authors:** Yelena P Wu, Jonathan J Chipman, Leighann Kolp, Tammy K Stump, Tatyana V Kuzmenko, Guilherme Del Fiol, Benjamin Haaland, Kimberly A Kaphingst, Roger Brooks, Adam L Hersh, Hannah L Brady, Kelly J Lundberg, Neng Wan, Courtney Carroll, Brian Orleans, Jennifer Wirth, David W Wetter

**Affiliations:** 1 Department of Dermatology School of Medicine University of Utah Salt Lake City, UT United States; 2 Huntsman Cancer Institute Salt Lake City, UT United States; 3 Department of Population Health Sciences School of Medicine University of Utah Salt Lake City, UT United States; 4 Department of Biomedical Informatics School of Medicine University of Utah Salt Lake City, UT United States; 5 Department of Communication College of Humanities University of Utah Salt Lake City, UT United States; 6 Granite School District South Salt Lake City, UT United States; 7 Department of Pediatrics School of Medicine University of Utah Salt Lake City, UT United States; 8 Department of Psychiatry School of Medicine University of Utah Salt Lake City, UT United States; 9 School of Environment, Society, and Sustainability College of Social and Behavioral Science University of Utah Salt Lake City, UT United States

**Keywords:** public health, school health services, preventive health services, COVID-19 testing, communicable disease control, text messaging, mHealth

## Abstract

**Background:**

A key challenge for schools throughout the COVID-19 pandemic was finding ways to monitor and prevent COVID-19 cases. While diagnostic testing and connecting students and their families to appropriate resources to mitigate the spread of COVID-19 were recommended, few schools had scalable infrastructure, including information technology systems, to implement these types of measures.

**Objective:**

This study tested a new approach to COVID-19 testing (SCALE-UP Counts) in school settings that used automated bidirectional text messages provided to the school community that alerted parents of students to COVID-19 testing options and guidance on when to test.

**Methods:**

The SCALE-UP Counts trial was designed as a Sequential Multiple Assignment Randomized Trial and final analyses compared results from parents who received intensive, fully automated, bidirectional text messaging about COVID-19 testing or usual care (control; fully automated unidirectional text messaging about COVID-19 testing), unblinded interventions. From the 16 selected schools, we enrolled all eligible participants who did not opt out of the study. The study provided schools from both arms of the trial with free at-home COVID-19 test kits. The primary outcome was the proportion of parents whose households tested for COVID-19, and the secondary outcome was the number of missed school days. The study asked parents to respond to self-report measures on testing outcomes and missed school days through web-based questionnaires.

**Results:**

The study included 7122 parents of students from 16 schools, half of which were title 1 schools; 2588 were randomized to usual care or control and 4534 to bidirectional text messaging. The SCALE-UP Counts intervention led to increased self-reported testing when compared with the control condition (22.8% vs 13.5%, relative testing rate=1.64, 95% CI 1.31-2.02; *P*<.001). There was no observed difference in missed school days between the study arms (0.43 per month vs 0.28 in usual care, relative missed days rate=1.55, 95% CI 0.98-2.45; *P*=.06).

**Conclusions:**

SCALE-UP Counts worked closely with schools and the state’s public health system to implement and test a scalable health information technology approach that delivered automated text messages to students’ parents around COVID-19 testing and provided access to free at-home test kits. Such an approach can help facilitate COVID-19 testing among school communities, including those that provide education and resources to students and their families from racial or ethnic minorities and with low socioeconomic status. Similar health information technology approaches could be used to increase ease of access to testing, reduce testing burden, and provide tailored information on health measures in school communities for a variety of illnesses or public health concerns.

**Trial Registration:**

ClinicalTrials.gov NCT05112900; http://clinicaltrials.gov/ct2/show/NCT05112900

## Introduction

### COVID-19 and Schools

Since the onset of the COVID-19 pandemic, there have been more than 6 million reported hospitalizations and more than 1 million deaths in the United States [1]. While originally thought to be primarily a disease affecting older adults, COVID-19 had far-reaching impacts across all age groups, with 15.6 million children in the United States having tested positive for COVID-19 by May 2023 [2]. Although most children experience mild or even asymptomatic infections, severe infections can occur as well as postinfectious complications, including multisystem inflammatory syndrome in children, which often causes critical illness [3,4]. Children from racial or ethnic minority groups and families with lower levels of socioeconomic status (SES) were disproportionately affected by the COVID-19 pandemic [5-9]. For instance, non-Hispanic Black and Hispanic or Latino school-aged students have been overrepresented in COVID-19 statistics for overall incidence as well as hospitalization for severe disease compared with all or non-Hispanic White students [5,6]. Rates of school absenteeism were also higher among non-Hispanic Black and Hispanic or Latino students than among White students [9]. For all children and their families, one of the most significant impacts of the COVID-19 pandemic was its effects on school systems.

A key challenge for schools throughout the pandemic was finding ways to monitor and prevent cases of COVID-19 to maintain in-person instruction, as well as other school-based services and resources (eg, meal services, primary care, and mental health services) that many children rely on [10,11]. Early in the pandemic, school closures impacted more than 50 million children in the United States and were associated with several adverse social and health outcomes for students, such as disparities in math and reading achievement, decreases in social connectedness, and increases in mental health conditions (ie, depression and anxiety) [6,9]. Local education agencies were encouraged by the Centers for Disease Control and Prevention and other federal organizations to communicate up-to-date COVID-19 information with students’ families, including information about testing resources. The Centers for Disease Control and Prevention also recommended providing diagnostic testing or connecting students and staff to appropriate resources in order to mitigate the spread of illness; however, few schools had scalable infrastructure, including information technology systems, available in place to implement these types of measures [12-14].

In the first phases of the COVID-19 pandemic, school-based testing programs included weekly surveillance testing for students and staff as well as symptomatic testing [15-19]. Many programs involved on-site testing at school or other district locations, which required community health workers, school nurses, or other staff to implement [15-17,19]. At the start of the COVID-19 pandemic, Utah was one of the first states to implement a “Test-to-Stay” model. This model required schoolwide COVID-19 testing for all students to continue attending classes in-person if at least 1.0% of the school’s population reported a positive COVID-19 diagnosis within 2 weeks [20,21]. Under this model, the state permitted students who tested negative to continue in-person instruction but required students who did not test or tested positive to quarantine or isolate and move to remote learning. During the 2021-2022 school year in Utah, the Utah Department of Health and Human Services reported 60,127 cases of COVID-19 among students K-5, 38,311 cases among students grades 6-8, 69,858 cases among students grades 9-12, and 1640 hospitalizations for K-12–age students (aged 5-17 years) statewide [22]. One of the school districts participating in this study had the third highest number of cases in the state during the 2020-2021 school year.

While implementation of school-based and at-home testing was associated with increased testing among students, parents, and school staff, several barriers led to lower-than-expected participation rates in testing programs. For instance, some parents and staff were reluctant to test themselves or their children due to knowledge gaps, potential pain involved in testing, misinformation about the COVID-19 virus, concerns about delay in receiving test results and receiving false-positive test results, concerns about privacy with contact-tracing procedures or reporting of test results, communication barriers (ie, language barriers and lower health literacy), and lack of awareness of available resources [16,17,23]. Several studies assessed the feasibility of both at-home and on-site COVID-19 testing to address the barrier of access to testing resources. Results indicated that both at-home and school-based surveillance, screening, and symptomatic testing were effective in decreasing infection rates and missed school days, especially for vulnerable populations (eg, rural settings, low SES, single-parent households, and racial or ethnic minorities) with otherwise limited access to testing resources. However, school-based testing emerged as the preferred method due to concerns about adequate support to ensure participation and consistency with at-home testing [15,17-19].

### Rationale for the SCALE-UP Counts eHealth Intervention

This study tested a new approach to COVID-19 testing in school settings that aimed to address these barriers using a stepped approach that used automated bidirectional text messaging (TM) provided to the school community that alerted parents to the availability of free, rapid COVID-19 tests, provided guidance on when to test, and, for a subgroup of those who needed it, provided access to a health navigator who could address motivations to test, logistical barriers to testing, and questions about COVID-19 testing. In addressing these barriers, the ultimate goals were to increase testing and decrease the number of missed school days. Several studies have used similar stepped-care approaches (ie, TM, followed by access to a navigator) to address mental health care, HIV support and treatment, and chronic disease (eg, diabetes, heart disease, and cancer) prevention and management among lower SES, minority, and other vulnerable populations. These digital health interventions, combined with navigation, can increase connection to care and improve health outcomes compared with standard care alternatives [24-30]. Studies using health navigator services have shown that access to a navigator can decrease health disparities by addressing barriers and concerns regarding health care access [31,32]. This study compared these interventions with a control intervention that used unidirectional TM. We selected this control intervention because it controlled for the modality of TM, reflected more typical methods of public health messaging, and allowed participants allocated to the control group to receive relevant COVID-19 testing information.

### Study Goals and Hypotheses

SCALE-UP Counts examined the efficacy of technology-supported interventions in promoting COVID-19 testing among K-12 school communities (students and staff). Interventions were informed by ongoing input from key stakeholders, including school staff, parents, the state department of health, and community organizations. The primary outcome of the trial was the proportion of parents whose households tested for COVID-19 (a relative testing rate [RTR] between study groups), and the secondary outcome was the number of missed school days. We hypothesized that the interventions in SCALE-UP Counts, when compared with a control intervention, would increase the proportion of students and school staff who tested for COVID-19 and would be related to the number of missed school days.

## Methods

### Participants, Setting, and Recruitment

Participants in this study included parents or legal guardians of students (termed “parents” for the remainder of this manuscript) and staff from 14 schools (elementary, middle, and high schools) within one of the largest school districts in Utah, plus 2 Utah charter schools [20-22]. In the 2020-2021 school year, 31.4% of schools in this district were title 1 schools, which received federal funding due to a high number or percentage of children from low-income families. We focused our recruitment efforts on title 1 schools. Parents were eligible to participate if they had a child or student attending a partnering elementary, middle, or high school; had a functioning cellular phone that could receive phone calls and text messages; and had the technology literacy necessary to send text messages. This analysis focuses on parent and child outcomes; however, school staff were also eligible to participate in the study.

The study received a waiver of documentation of consent from the institutional review board (IRB), allowing for expedited recruitment and enrollment. Parents were recruited through several methods, depending on whether their school or district used an opt-out or opt-in method for research studies; however, only the opt-out schools were included in this analysis. For schools using an opt-out method, parents were sent an initial Health Insurance Portability and Accountability Act–compliant bidirectional text message that described the study and stated its affiliation with the University of Utah, provided a link to the study website and consent form (both of which were branded with the institutional affiliation and included information on all the interventions being tested), and the opportunity to opt out of the program. The consent form on the study website was text-based but brief (approximately 300 words), written at a Flesch-Kincaid Grade Level of 7.4, and available in both English and Spanish. Next, they were sent a text message by the study team informing them that they would be receiving text messages regarding COVID-19 testing and could opt out at any time. Parents who did not opt out within 24 hours of receiving the text message were enrolled in the study. Schools also distributed and displayed study flyers (in English and Spanish) in person and via social media to provide parents additional information about the study.

### Design

**Figure 1:**
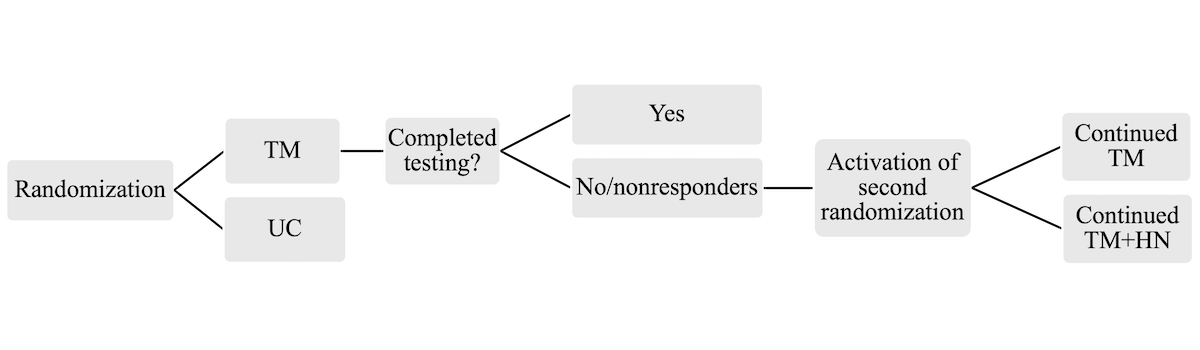
Study design. HN: health navigation; TM: text messaging; UC: usual care or control.

SCALE-UP Counts was a parallel study designed as a Sequential Multiple Assignment Randomized Trial. The Sequential Multiple Assignment Randomized Trial design was selected based on its demonstrated use for developing adaptive interventions that can have a large public health impact [33-35]. The overall study design is depicted in Figure 1 and includes 2 levels of randomization. The random allocation sequence was generated by the study statistician (JC) using R’s randomizeR package (R Project for Statistical Computing) [36] and implemented automatically by the in-house Digital Health to Advance Research Equity (DHARE) software (University of Utah Department of Biomedical Informatics), further described in the “Intervention Arm: TM and HN” subsection, which automatically assigns participants to study arms based on the randomization sequence produced by R. This enabled the allocation to be concealed from investigators and study team. All participants were assigned a first- and second-level randomization by the study statistician at the start of the study and were unblinded to the intervention. In the first stage, participants were randomized to intensive TM or usual care or control (UC) using an allocation ratio of 80.0% intensive TM and 20.0% UC using Soares and Wu’s Big Stick Design with a maximum tolerable imbalance of 3 to maintain the desired allocation ratio and to reduce the risk of chronological imbalances between treatment arms over time [37,38]. Participants who were assigned to intensive TM but did not report testing outcomes within any TM cycle (approximately 28-30 days per cycle) when they had previously indicated that they would test were considered “nonresponders.”

Nonresponding participants transitioned to their second stage of randomization, which consisted of either continued intensive TM or intensive TM plus health navigation (HN) (intensive text messaging plus health navigation [TM+HN]) with an allocation ratio of 50.0% TM and 50.0% TM+HN. The HN component followed a validated behavior change approach [39]. The allocation ratio for the first randomization was updated to 50.0% TM and 50.0% UC [40] after the first year of the study. This update was made to optimize statistical power to be able to compare intensive TM versus UC intervention arms in end-of-study analyses, because the self-report rate in the first year (20.0%) was much lower than anticipated. This would impact our ability to compare TM with TM+HN. Changes from the original study design and sample size considerations are documented in the statistical analysis plan (Multimedia Appendix 1). Because of the low self-report rate, it became clear that sensitivity analyses would be critical. Throughout the study, the power to detect small to moderate effect sizes was reassessed while making the most conservative assumptions about missing data. The end sample size of 7122 was determined based upon simulations, which were more than 80.0% powered to detect a treatment effect of 0.30 among self-reported outcomes and conjectured an equal testing rate among unreported outcomes (we considered this a conservative assumption).

### Test Kit Distribution

All parents, regardless of study arm, were provided access to at-home COVID-19 test kits, free of charge. Via text message, all parents were informed of test kit availability at school administration offices and family engagement centers that provided health-related and other social services to school communities. Parents were permitted to obtain test kits for themselves as well as any household members.

### UC Arm

Participants randomized to UC received unidirectional, static text messages every 3 weeks that mirrored public health messages about COVID-19 testing, such as recommendations to test for COVID-19, offering tests, and testing options through the school and district or via direct mailing through the research project, which could be requested via email.

### Intervention Arm: TM and HN

Participants randomized to intensive TM were sent bidirectional text messages related to COVID-19 testing. Additional information on frequency and content of text messages is described in the following paragraphs. We used the DHARE platform for TM, which was developed by coauthor Dr Del Fiol and other members of the ReImagine EHR program of the University of Utah Department of Biomedical Informatics. DHARE has been supported by multiple grants from the National Institutes of Health, awarded to investigators from the University of Utah Department of Biomedical Informatics and Huntsman Cancer Institute. DHARE is a scalable, open-source software platform to create digital health interventions using bidirectional TM. DHARE enables the creation of multilevel TM conversation flows that prompt for single-word responses and provides a web-based dashboard for staff to track patient outreach activities. DHARE has supported the creation and implementation of digital health interventions in 7 pragmatic trials addressing health equity in collaboration with 13 Utah Community Health Center systems and their approximately 40 primary care clinics, and with Medicaid patients served by University of Utah Health [24,41-44]. The messaging for this trial was based on a prior trial that used DHARE to deliver a COVID-19–testing text message intervention [43,44], with content informed by the most recent state and federal public health guidance as well as a pediatric infectious disease specialist. Due to changing COVID-19 testing public health guidance over the course of the study, approximately 2 months into the study, intervention messaging shifted away from COVID-19 testing for diagnostic purposes and toward testing for both diagnostic and preventative purposes. All messaging was fully automated and synchronous. All phone numbers were first verified using DHARE to ensure that each participant’s phone number could receive text messages. In total, 81 out of 7813 (1.0%) potential participants did not have a valid phone number and were unable to receive text messages, so they were excluded from the study.

Intensive TM participants were sent bidirectional text messages related to COVID-19 testing, where the participant could engage with messaging. Each “cycle” of TM lasted for 28-30 days (variation in lengths due to changing testing recommendations during the pandemic), with the intervention lasting for 21-23 days and the assessment lasting for 7 days. The number of text messages sent to each participant per cycle varied from 3 to 13 and was dependent on the participant’s responses, with the intervention consisting of 2-8 text messages (if the participant did not respond to the first intervention message, it was sent a second time) and the assessment consisting of 1-5 text messages. Each participant received multiple cycles of messaging during the research study, regardless of whether they requested COVID-19 tests or reported COVID-19 testing in prior cycles. The messaging cycle of the study’s longest running text message campaign (May 2022 to June 2023) is shown in Multimedia Appendix 2.

The first phase of each cycle (denoted as phase 1 in Multimedia Appendix 2) assessed testing needs, provided information on how to obtain test kits (through the school and district or via direct mailing through the research project, which could be requested through text message), and assessed whether testing would be completed over the next 7 days. The second phase of each cycle (denoted as phase 2 in Multimedia Appendix 2) assessed whether testing was completed for those who indicated that they would test within the next 7 days and determined whether participants could receive additional intervention for COVID-19 testing based on their pattern of responses. Participants received their phase 2 condition of continued intensive TM or intensive TM+HN if they replied “yes” to the screener question about testing but replied later that they did not test or did not reply with any testing outcome.

In phase 2, continued intensive TM consisted of text messages with information on the importance of testing, prompts for testing, and testing options (community testing, test kit pickup, and request a mailed test kit). Each cycle of intensive TM+HN could also include 1 telephone call from 1 of 2 health navigators focused on addressing testing barriers and providing information on accessing testing. TM+HN participants who had been sent a text message recommending a COVID-19 test but who did not report completing a test were placed in a call queue. We recognized that convenient times of the day to receive a call may differ between participants, so calls were made at a variety of times of day (7:30 AM to 10:30 AM, 10:30 AM to 3 PM, and 3 PM to 6 PM). If a participant did not answer a call, the participant was called the next day during a different time slot. If that call was also missed, a final attempt was made the following day during the last time slot that a navigator had not yet used for that participant. Navigators completed case management training and were trained in an empirically validated behavior change approach (Motivation and Problem Solving) [25,39,45-51]. Text messages and HN services were available in English and Spanish.

### Measures

#### Deidentified Demographic and Testing Information

Deidentified data were obtained to characterize the study sample demographics and are provided for descriptive purposes. Deidentified student-level data were provided by the largest participating school district. These data included the race and gender of students, the preferred language of parents who participated in the study, and the percentage of students eligible for free or reduced lunch.

#### Self-Reported Data

Parents completed assessments about themselves, their household, and enrolled children. Assessments were completed through assessment phase text message cycles and monthly web-based surveys, both of which collected information on the intervention’s primary outcome of COVID-19 testing and secondary outcome of missed days of school. All assessments were available in both English and Spanish. The entire text message cycle for all intervention groups was approximately 30 days, with the intervention cycle lasting for 23 of those days and the assessment cycle lasting for 7 of those days.

The web-based surveys were built and deployed through REDCap (Research Electronic Data Capture) [52], a secure web platform for building surveys. Each parent was sent a link to the monthly survey via email. As closed surveys, only those who were sent a link had access to the surveys. Each survey was branded with the institutional affiliation. The number of items on the survey varied between 2 and 22, depending on the number of children in the family and whether any family members completed COVID-19 testing. The items were nonrandomized, and they were split across multiple pages; a button allowed survey takers to return to previous pages of the survey to change their answers. The REDCap system records all data, even if the survey taker does not press the submission button. As a result, incomplete surveys could be “submitted.” The items reported on were not specifically validated for web-based use and were drawn from the National Institutes of Health Rapid Acceleration of Diagnostics Underserved Populations (RADx-UP) Common Data Elements (CDEs). The CDEs were derived from sources including the National Institutes of Health CDE Repository [53], Disaster Research Response guidelines [54], and the PHENotypes and eXposures Toolkit.

#### Testing Outcomes

Parent participants provided information on their COVID-19 testing. Assessment phase text message cycles collected information on the number of household members tested, test results for adults and children, and names of children who tested positive during the intervention cycle. The primary testing outcome was whether any household members used a COVID-19 test in the previous 30 days, as reported during the assessment phase text messages.

#### Missed School Days

The number of missed school days for students was collected through web-based REDCap surveys delivered monthly. In the surveys, parents were asked to report the number of school days their children missed due to COVID-19 symptoms, exposure, quarantine, and testing. All available data on missed days were used in the analysis, regardless of whether the rest of the survey was completed. The secondary outcome was the average number of monthly days missed per participating child.

#### Social Determinants of Health

Social determinants of health (SDOH) factors were determined by geocoding parents’ home addresses and linking them with census tract-level sociodemographic factors using ArcGIS Pro [55]. For each parent, the Social Vulnerability Index (SVI), which has been used as a measure of endurance of communities encountering stressors, including disasters and health problems [56], was calculated from 16 single SDOH variables reflecting that community (eg, SES, household characteristics, racial and ethnic minority status, housing type, and transportation). We used the 2017-2021 American Community Survey 5-year data for the 2023 SVI values [56] in this study. SVI scores range from 0 to 1, with 0 being the least vulnerable and a score of 1 being the most vulnerable to stressors or most disadvantaged. Because SVI is a ranked-based measure, we used below versus above 0.5 as scientifically meaningful subgroups for analysis. SVI is one of the most frequently used SDOH measures across health-related studies, including COVID-19 infection [57-62], and was included in this study, given the disproportionate impact of COVID-19 infection on disadvantaged communities and this study’s goal of serving these communities.

### Analytic Plan

Our primary summary measure of interest was the RTR between those randomized to TM versus UC in the first stage of randomization, and the secondary summary measure of interest was the relative missed days rate (RMD 30) during the academic school year between the same treatment arms of interest. As mentioned in the “Design” section earlier, the summary measure of interest changed during enrollment because the rate of participants qualifying as nonresponders and the overall self-report rate were too low to make meaningful comparisons between TM and TM+HN. The summary measures were estimated, allowing for the TM effect to vary over calendar time. The statistical analysis plan and corresponding analysis, along with the full trial protocol, are detailed at ClinicalTrials.gov (NCT05112900). Multimedia Appendix 1 includes an update to the analysis plan, which further includes the interim analysis power calculations and the SVI subgroup analysis plan. The outcome assessors and data analysts were not blinded to which intervention was the intervention of interest. For analyses that used SVI or the number of enrolled children, missing data were imputed using mean imputation conditioned on school enrollment.

RTR was estimated separately for each era of allocation enrollment (ie, 80.0% TM vs 20.0% UC and then 50.0% TM vs 50.0% UC) and then aggregated as a meta-analysis [40]. This was important to remove potential bias in the treatment effect [40]. Since students were enrolled in school batches, the overall TM arm would disproportionately reflect earlier enrolled schools than the UC arm. Analyzing each allocation era separately ensures a fair comparison across arms. For each allocation era, we used generalized estimating equations with a logit link to estimate the treatment-specific rate of testing over follow-up calendar time. We clustered by participant and used an exchangeable working model with robust standard errors. The model included treatment, calendar date modeled with restricted cubic splines having 5 knots [63], and their interaction. The testing rate for each arm was estimated by averaging over the predicted testing rate from the earliest to the last calendar day of the allocation era’s follow-up. We aggregated results across allocation eras by inverse variance weighting the log RTR and back-transformed to estimate the RTR [64]. We used bootstrap sampling to estimate the variability in estimating the log RTR, which we then used to calculate 95% CIs and statistical significance for the back-transformed RTR. The primary analysis was performed using observed outcomes, which are approximately unbiased when the missing at random (MAR) assumption holds [65]. However, we conducted several sensitivity analyses to account for the low self-report rate of outcomes; 35.3% (2517/7122) of participants self-reported on testing at least once, 19.8% (9698/48,878) of all possible testing outcomes were self-reported, 9.1% (642/7021) of participants self-reported missed school days, and 6% (2553/42,538) of all missed school day outcomes were self-reported.

The first sensitivity analysis used a pattern-mixture model [66] with the assumption that outcomes were predictable from observed baseline and nonbaseline covariates (ie, MAR). A model for each treatment arm was fit adjusting for calendar date modeled with restricted cubic splines having 5 knots, parent type (mother, father, and other), number of enrolled children, SVI with restricted cubic splines having 5 knots, whether or not the participant opted out early (by the fifth assessment), the participant’s observed self-report rate, and the interaction between early opt-out and self-report rate. For each arm, the testing rate throughout allocation follow-up was estimated for each participant, and the overall testing rate was then averaged over each participant’s predicted testing rate. We also performed tipping point analyses that conjectured (ie, not assuming MAR) what the RTR would need to be to nullify results. We used the pattern-mixture model as the base model and conjectured the testing rate to be a fraction of the participant’s predicted rate when outcomes were not self-reported. We varied the fraction under both arms. As 2 extremes, we assumed that testing either never or always occurred when participants did not self-report on testing.

Our secondary summary measure of interest, RMD 30, was estimated following a similar strategy as the RTR; however, we used a Gaussian generalized estimating equations link rather than a logit link. Also, we used only self-reported outcomes that occurred when school was in session between August 2022 and May 2023. We included schools that were in session during these months; 1 school that was in session between April and November was excluded. The leading analysis used observed outcomes. We performed a similar suite of sensitivity analyses using the pattern-mixture model and tipping point analyses. For the pattern-mixture model to converge for each treatment arm, the model was simplified to adjust for calendar date modeled with restricted cubic splines having 5 knots, parent type (mother, father, and other), number of enrolled children, SVI below versus above 0.5, and the participant’s observed self-report rate. We also used the school district’s attendance rate in the 2023 academic year to conjecture the missed school days when unreported [67].

For both RTR and RMD 30, we tested for an SVI effect modification. Following a similar strategy described for RTR and RMD 30, we estimated and tested for any added (or diminished) treatment effect when receiving TM between subgroups. Regardless of statistical significance, we provided estimates for the TM effect within both subgroups. We assessed for effect modification under observed outcomes and the pattern-mixture model.

### Ethical Considerations

This study was approved by the University of Utah IRB (IRB_00143340). The study received a waiver of documentation of consent from the IRB, allowing for expedited recruitment and enrollment. All participants were given the contact information of the research team and were able to contact them by text, phone, or email with any safety or security concerns. The privacy and confidentiality of all participants’ data were maintained using REDCap [52], a secure web-based platform for building, dispensing, and collecting information from surveys. All data were anonymized prior to analysis. Opportunities for compensation were provided to the participants to encourage participation and retention. All parents who completed both the assessment phase text messages and the survey in a particular month were entered into that month’s raffle for a US $100 gift card. In addition, participants in the study could receive compensation for completing other assessments not included in this analysis. The results of this study are reported in accordance with CONSORT (Consolidated Standards of Reporting Trials) guidelines for web-based and mobile health interventions (Multimedia Appendix 3) [68].

## Results

### User Statistics

**Figure 2:**
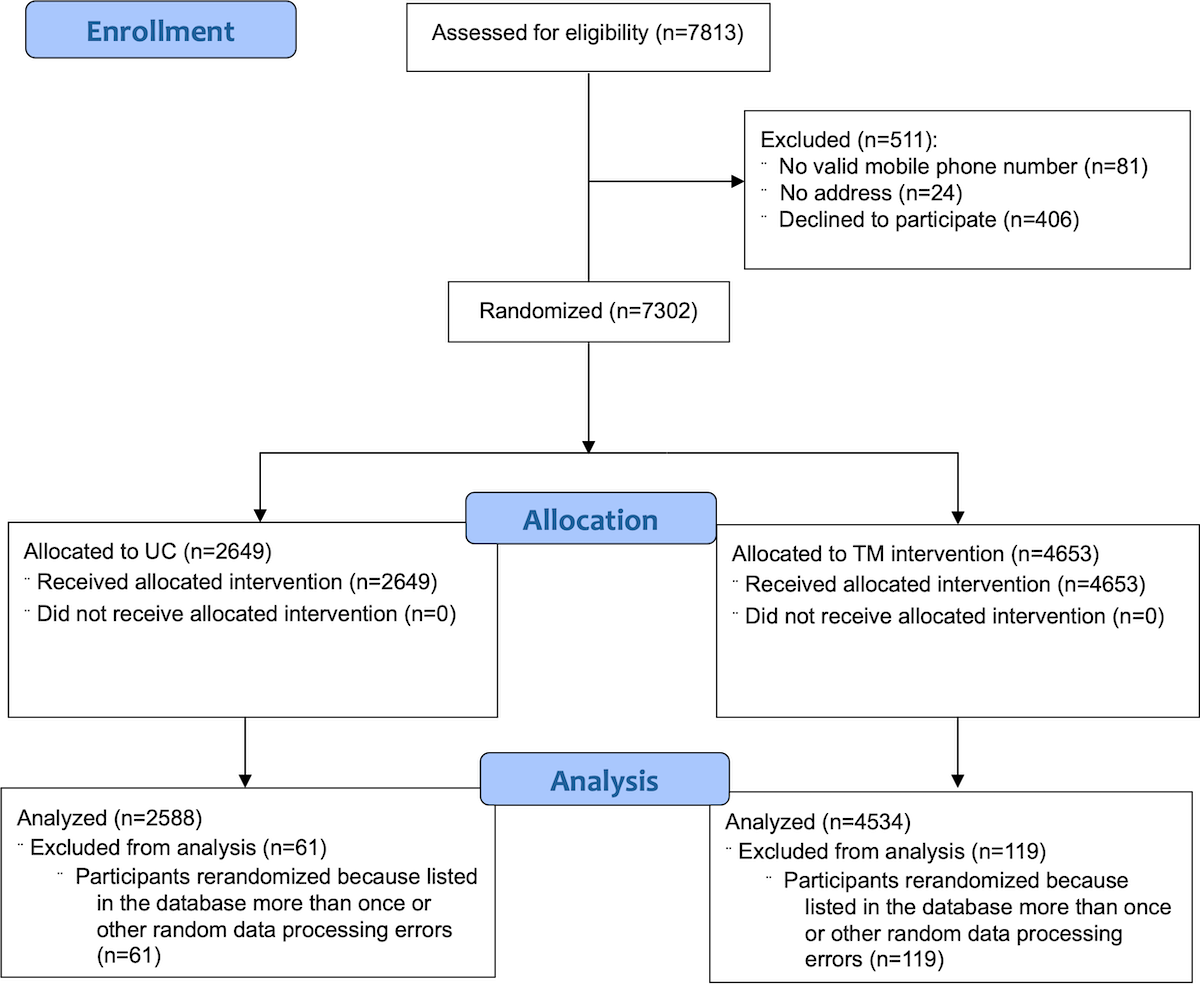
CONSORT (Consolidated Standards of Reporting Trials) flow diagram. TM: text messaging; UC: usual care or control.

User statistics are shown in Figure 2. This analysis focuses on 7122 participants. Out of 7302 eligible participants, DHARE randomly assigned 2649 to the UC arm and 4653 to the TM arm, and each received the initial text message. DHARE randomized 61 participants in the UC arm and 119 participants in the TM arm a second time and sent a second initial text message due to a technical error (eg, being listed more than once in the database); we excluded these individuals from analysis, leaving 2588 UC participants and 4534 TM participants in the analysis set. The study recruited participants between February 2022 and May 2023, began follow-up of outcomes in March 2022, and ended follow-up in June 2023. This study concluded when the 2022-2023 school year ended, near the end of the project’s funding period.

Half (8/16, 50.0%) of the participating schools were title 1 schools (schools that receive federal funding due to a high number or percentage of children from low-income families), and less than half (3840/9633, 39.9%) of the student body was eligible for free or reduced lunch according to school-level data for the 2021-2022 school year. SVI for students across all 16 schools had a range of 0.0014-1, with a mean score of 0.58 (median 0.58), indicating that, on average, students in the schools served are more disadvantaged than the state average of 0.50 [56]. Table 1 shows demographic characteristics of students from the largest participating school district (14 schools) based on data provided by the school district. The student gender distribution was approximately equal (3364/6803, 49.4% female and 3431/6803, 50.4% male). Most of the students in the schools were White (3967/6803, 58.3%), and approximately one-quarter (1924/6803, 28.3%) were Hispanic. English was the preferred language for the majority (4962/6803, 72.9%) of students, with almost one-quarter (1449/6803, 21.3%) having Spanish as their preferred language. Most of the participating parents were female (5340/7122, 75.0%). On average, participants received 7 opportunities to self-report (IQR 2-10 opportunities).

**Table 1:** Demographic characteristics of students from the largest participating school district whose parents or guardians participated in this study.

Characteristic		UC^a^	TM^b^	Total^c^
**Sex, n (%)**				
	Male	1197 (50.6)	2191 (50.3)	3431 (50.4)
	Female	1166 (49.3)	2158 (49.6)	3364 (49.4)
	Unknown	4 (0.2)	4 (0.1)	8 (0.0)
**Race or ethnicity, n (%)**				
	African American	89 (3.8)	156 (3.6)	246 (3.6)
	American Indian/Alaskan Native	17 (0.7)	58 (1.3)	78 (1.1)
	Asian	108 (4.6)	254 (5.8)	365 (5.4)
	Caucasian/White	1329 (56.1)	2593 (59.6)	3967 (58.3)
	Hispanic/Spanish	752 (31.8)	1147 (26.3)	1924 (28.3)
	Pacific Islander	69 (2.9)	142 (3.3)	217 (3.2)
	Unknown	3 (0.1)	3 (0.1)	6 (0.0)
**Preferred language, n (%)**				
	English	1657 (70.0)	3249 (74.6)	4962 (72.9)
	Spanish	579 (24.5)	846 (19.4)	1449 (21.3)
	Other	131 (5.5)	258 (5.9)	392 (5.8)
Free or reduced lunch (% of student body by school), mean (range)		N/A^d^	N/A^d^	53.5 (14.7-87.2)
Social Vulnerability Index,^e^ mean (range)		0.62 (0.0071-1)	0.59 (0.0014-1)	0.60 (0.0014-1)

^a^UC: usual care or control.

^b^TM: intensive text messaging.

^c^Total column values exceed the sum of the UC and TM columns because 83 parents or guardians were randomized multiple times due to a technical error.

^d^N/A: not applicable.

^e^The Social Vulnerability Index is a composite measure of SDOH for each participant’s community based on their home address.

### Evaluation Outcomes

#### Testing Rate

**Figure 3:**
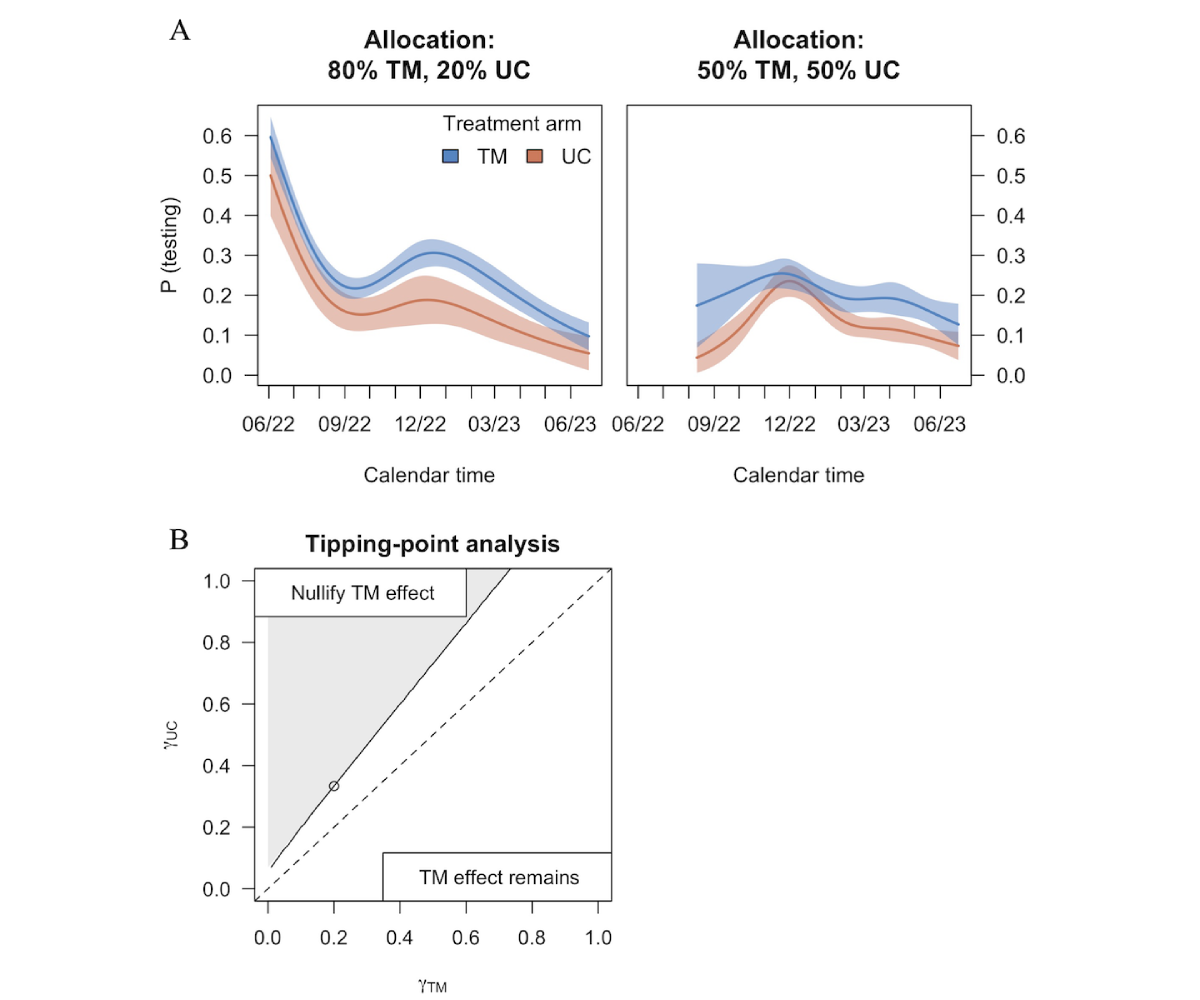
Testing rates based on allocation era (A) and sensitivity analysis for testing rates (B). TM: text messaging; UC: usual care or control.

Aggregating across allocation eras, the testing rate was 1.5 times higher in the TM arm than in the UC arm (21.6% vs 14.4%, RTR=1.50, 95% CI 1.35-1.67; *P*<.001; Table 2 and Figure 3A). This estimated effect was robust to sensitivity analyses. Under the pattern-mixture model sensitivity analysis, the testing rate aggregated across allocation eras was 22.8% in the TM arm versus 13.5% in the UC arm (RTR=1.64, 95% CI 1.31-2.02; *P*<.001). In one extreme, if unreported testing outcomes were indicative of never testing, the RTR still favored the TM arm (4.2% vs 2.9%, RTR=1.46, 95% CI 1.29-1.65; *P*<.001). In the other extreme, where we assume that testing always occurred among unreported testing outcomes, the RTR continued to favor the TM arm (84.7% vs 82.8%, RTR=1.02, 95% CI 1.01-1.04; *P*=.002). To further understand the impact of unreported outcomes, Figure 3B provides scenarios of testing rates among unreported outcomes which would nullify the TM effect. For example, the TM effect would be nullified if there were a testing rate of 33% among UC unreported outcomes versus 20% among TM unreported outcomes. We did not observe differential effects by SVI subgroups (Table 3; *P=.*20 using observed outcomes and *P*=.47 using the pattern-mixture model).

**Table 2:** Relative testing rate of intensive text messaging versus usual care or control and sensitivity analyses (average treatment effect).

Test	RTR^a^	95% CI	*P* value
Primary analysis (observed outcomes)	1.50	1.35-1.67	<.001
Pattern-mixture model (MAR^b^)	1.64	1.31-2.06	<.001
Missing conjecture: no testers	1.46	1.29-1.65	<.001
Missing conjecture: all testers	1.02	1.01-1.04	.002

^a^RTR: relative testing rate.

^b^MAR: missing at random.

**Table 3:** Relative testing rate of intensive text messaging versus usual care or control and sensitivity analyses (effect within Social Vulnerability Index levels)^a^.

Test		RTR^b^	95% CI	*P* value	Interaction test (*P* value)	
**Observed outcomes**						.20
	SVI^c^ <50	1.42	1.24-1.63	<.001		
	SVI 50+	1.62	1.38-1.91	<.001		
**Pattern-mixture model**						.47
	SVI < 50	1.56	1.20-2.02	.001		
	SVI 50+	1.72	1.35-2.20	<.001		

^a^See Table S1 in Multimedia Appendix 4 for estimates and tests for participants enrolled under each allocation era.

^b^RTR: relative testing rate.

^c^SVI: Social Vulnerability Index.

#### Missed School Days

**Figure 4:**
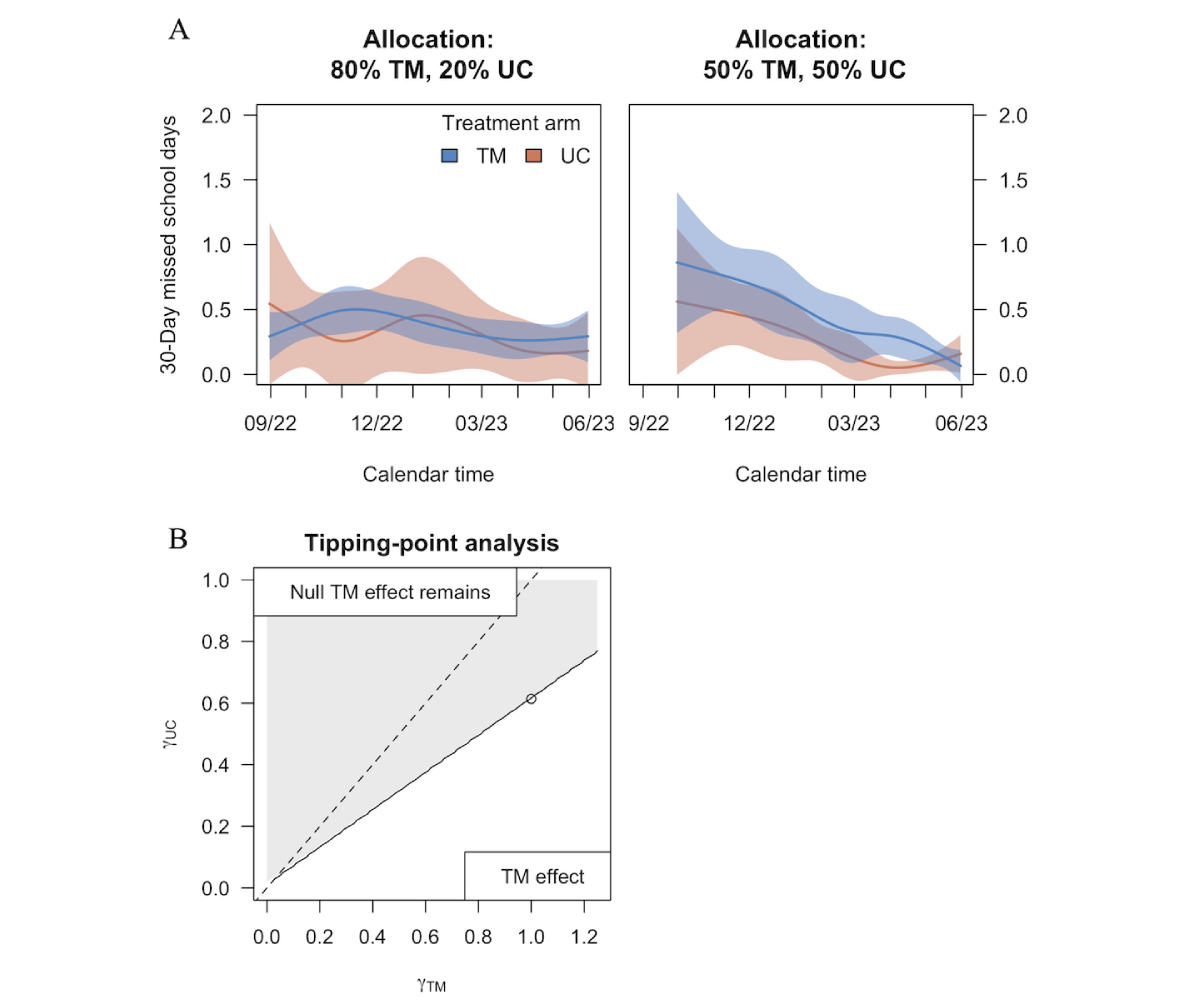
Missed school days by allocation era (A) and sensitivity analysis (B). TM: text messaging; UC: usual care or control.

Aggregating across allocation eras, the analysis showed no significant difference in the number of school days between UC and TM; there was insufficient evidence to observe a difference in the 30-day missed school rate (0.43 per month vs 0.28 in UC, RMD 30=1.55, 95% CI 0.98-2.45; *P*=.06; Table 4 and Figure 4A). Under the pattern-mixture model sensitivity analysis, the effect of the TM upon relative missed days rate showed a trend but was even less pronounced (0.67 per month in TM vs 0.58 in UC, RMD 30=1.24, 95% CI 0.65-2.39; *P*=.52). If we assume that the missed school days among unreported outcomes were consistent with the attendance reported by the school, the estimated effect further moves toward the null (1.71 in both arms, RMD 30=1.00, 95% CI 0.98-1.02; *P*=.87), which follows from the extent of data missingness for self-reported outcomes. To observe an increase in TM missed school days, Figure 4B provides scenarios for which the unreported school days among UC and TM would be enough to observe a TM effect (eg, TM missing 1 day on average vs UC missing 0.61 days on average). We did not observe differential effects by SVI subgroups (Table 5; *P=.*86 using observed outcomes and *P=*.67 using the pattern-mixture model).

**Table 4:** Relative missed school days rate (RMD 30) of intensive text messaging versus usual care or control and sensitivity analyses (average treatment effect).

Test	RMD^a^ 30	95% CI	*P* value
Primary analysis (observed outcomes)	1.55	0.98-2.45	.06
Pattern-mixture model (MAR)^b^	1.24	0.65-2.39	.52
Missing conjecture: reported school rate	1.00	0.98-1.02	.87

^a^RMD 30: relative missed days rate.

^b^MAR: missing at random.

**Table 5:** Relative missed school days rate (RMD 30) of intensive text messaging versus usual care or control and sensitivity analyses (effect within Social Vulnerability Index levels)^a^.

Test		RMD 30^b^	95% CI	*P* value	Interaction test (*P* value)	
**Observed outcomes**						.86
	SVI^c^ <50	1.58	0.89-2.79	.12		
	SVI 50+	1.70	0.89-3.27	.11		
**Pattern-mixture model**						.67
	SVI <50	1.09	0.56-2.14	.80		
	SVI 50+	1.35	0.67-2.73	.41		

^a^See Table S2 in Multimedia Appendix 4 for estimates and tests for participants enrolled under each allocation era.

^b^RMD 30: relative missed days rate.

^c^SVI: Social Vulnerability Index.

## Discussion

### Principal Results

We conducted SCALE-UP Counts among predominantly underserved school communities, and results indicated that a bidirectional TM intervention, which offered an easy and convenient way to order COVID-19 tests, led to increased self-reported testing compared with fewer noninteractive (ie, 1-way) text messages related to COVID-19 testing. To our knowledge, this is the first study to examine the impact of a text message–based intervention to deliver rapid, at-home COVID-19 testing options to multiple school communities. The vast majority of approaches to COVID-19 testing during the earlier phases of the pandemic relied on on-site testing at schools [69-71], with similar approaches used to promote COVID-19 vaccination [72]. Our finding that the SCALE-UP Counts TM intervention promoted reported COVID-19 testing is consistent with studies showing that such interventions can improve implementation of a range of desired health behaviors, including attendance at medical appointments and medication adherence [73,74]. Furthermore, we did not observe differences in the benefits of reported COVID-19 testing by SDOH. Individuals from a range of SESs were able to benefit from the TM intervention. This finding is notable, given the literature documenting that individuals living in more socioeconomically disadvantaged neighborhoods, as well as minority ethnic groups, have higher rates of almost all of the known underlying clinical risk factors that increase the severity of and subsequent mortality due to COVID-19 [75-79]. A relatively easy-to-access intervention, such as TM, together with the availability of at-home resources (ie, test kits in this case), could be a useful type of intervention for underresourced individuals and communities.

In terms of missed school days, there could be 2 possible outcomes of COVID-19 testing. First, it could potentially reduce missed days by confirming that symptoms were not related to COVID-19 or that a student did not need to isolate after an exposure; second, it could potentially increase missed days by detecting cases and thus limiting school transmission. This study did not observe a difference in missed school days between the study arms, suggesting that access to testing did not negatively impact school attendance, although missed school days tended to be higher in the bidirectional texting arm. Future studies that have access to school attendance records could help address the challenges of examining this outcome with limited self-reported data.

In the context of this trial, the implementation of the intervention and trial procedures involved software programmers who could set up separate study arms and modify the content of the text messages based on the phase of the pandemic and the latest COVID-19 guidance. Outside of this trial setting and when certain study design elements (eg, separate study arms) are not necessarily required, school systems could use preexisting TM systems and ideally modify the text messages based on current public health guidance. The intervention could also be implemented using TM systems available on the market, via the platform used in the current trial, which is open source, and could be modified if other systems were available. For instance, other strategies to apply interventions similar to SCALE-UP Counts in a public health context for other health-relevant events (eg, outbreak reporting, public health notifications, and guidance about immunizations) could use different forms of communication, such as email, social media, and surveys, that included embedded links for additional information.

### Strengths and Limitations

Strengths of this study included its use of an intervention modality (ie, TM) that is widely available, including among underserved communities. For instance, 95.0% of individuals with an annual income of less than US $30,000 and 96.0% with educational attainment of high school or less report having a phone that can text [80]. Half of all schools participating in this study were title 1 schools, and nearly half of the students were eligible for free or reduced lunch. The study findings are therefore relevant to disadvantaged or underserved populations. In addition, SCALE-UP Counts was a population-level intervention with potential for scalability, and the intervention was deployed in a relatively large sample size of schools. Despite the low self-report rate, the low-cost scalability allowed for increasing the sample size to draw conclusions robust to the most conservative missing data assumptions. Finally, this study included outcomes that are highly relevant to the goal of COVID-19 prevention (ie, testing) and to mitigating the impact of COVID-19 on individuals’ lives (ie, avoiding missed school days). While the population-level focus of SCALE-UP Counts was a strength, a limitation to this approach involves the challenges of capturing and maintaining research engagement in a community sample, including through individuals opting out of the study initially or over time and missingness of longitudinal research surveys especially as individuals experienced “testing fatigue” in the middle to later phases of the pandemic. Those who provided data on self-reported outcomes may represent a self-selected sample. For example, most parents who provided outcomes were female, which has implications for the generalizability of the results. Encouragingly, the results of this study appear to be robust to various assumptions regarding missingness, as seen in the sensitivity analyses. To facilitate participation and engagement of individuals who are not fluent in English or Spanish, consent forms and website materials for future studies could use simpler English, include graphics to aid understanding, and be offered in additional languages. Other limitations include the reliance on self-reported data and focus on a single geographic area, which limits its generalizability.

### Conclusions

In summary, SCALE-UP Counts worked closely with schools and the Utah public health system to implement and test a scalable health information technology approach that delivered automated text messages to students’ parents around COVID-19 testing and provided access to free at-home test kits. The results indicate that such an approach can help facilitate COVID-19 testing among school communities, including those that provide education and resources to students and their families from racial or ethnic minorities and with low SES. School systems could use similar health information technology approaches to increase ease of access to testing, reduce testing burden, and provide tailored information on health measures in school communities for a variety of illnesses or public health concerns.

## Appendix

Multimedia Appendix 1 SCALE-UP Counts statistical analysis plan.

Multimedia Appendix 2 Text message intervention and assessment cycle. HN: health navigation; TM: text messaging.

Multimedia Appendix 3 CONSORT-EHEALTH checklist.

Multimedia Appendix 4 Outcomes for participants enrolled under each allocation era; effect within Social Vulnerability Index levels.

## Abbreviations

CDE: Common Data Element
CONSORT: Consolidated Standards of Reporting Trials
DHARE: Digital Health to Advance Research Equity
HN: health navigation
IRB: institutional review board
MAR: missing at random
RADx: Rapid Acceleration of Diagnostics
REDCap: Research Electronic Data Capture
RMD 30: relative missed days rate
RTR: relative testing rate
SDOH: social determinants of health
SES: socioeconomic status
SVI: Social Vulnerability Index
TM: text messaging
TM+HN: text messaging plus health navigation
UC: usual care or control

## Data Availability

The datasets for this manuscript are not yet publicly available because Rapid Acceleration of Diagnostics (RADx) data have not yet been made available to the public for review and analysis. Requests to access the datasets should be directed to the National Institutes of Health RADx Data Hub.
