# Supplementary material for: Mobile Intervention for Increasing COVID-19 Testing in K-12 Schools Serving Disadvantaged Communities: Randomized Controlled Trial of SCALE-UP Counts
Source: J Med Internet Res. 2025 Nov 11;27:e79775. doi: 10.2196/79775 (PMC12648121; doi:10.2196/79775)
Supplement: Multimedia Appendix 1 [file jmir_v27i1e79775_app1.pdf]

SCALE-UP Counts Statistical Analysis Plan

07/18/2024

Clinical Trial Registration: NCT05112900

Update: 04/02/2025 to include interim power calculations and SVI subgroup analysis plan

1 BACKGROUND

Starting in March 2020, most schools throughout the United States temporarily closed to mitigate the spread of the coronavirus disease 2019 pandemic (COVID-19). COVID-19 testing emerged as a critical tool for schools to determine when they could provide in-person instruction and activities. Yet, providing this service presented challenges, including the need for frequent testing events and/or test access, lack of staff time and training to provide testing guidance, and the need to respond to changes in COVID-19 testing and mitigation guidance. The SCALE-UP COUNTS study was designed to address key testing barriers faced within the school setting on when and how testing should be performed. The intervention was bi-directional text messaging (TM) to provide guidance and reminders designed to promote COVID-19 testing among K-12 school students and their families and school staff members. As the trial progressed, we observed a lower response rate to the testing outcome than anticipated. The purpose of this analysis plan is to describe the trial's original estimand of interest, disruptions to the trial design and updates to the estimand of interest, and the end statistical analysis plan. Working with key stakeholders was critical for adjusting to the COVID disruptions.

2 KEY STAKEHOLDERS

The Food and Drug Administration's published guidance on estimands [2], describes the importance of aligning the clinical trial estimand (quantity of interest) with the objectives of key stakeholders. Key stakeholders included the Utah Department of Health, the Granite and Salt Lake City School Districts, participating charter schoools, and the Principal Investigators of the grant proposal (Wu and Wetter).

3 ORIGINAL ESTIMAND (TM VS TM+MAPS):

The original objective was to test whether TM versus TM+HN would differentially (a) Increase the proportion of students tested out of those who meet the criteria for testing and (b) Decrease missed school days. The five key elements of the estimand included:

(1) Population: Families with students in the Granite and Salt Lake City School District and participating charter schools

(2) Treatment: Receipt of TM with or without a health navigator who would call and address barriers faced in testing
(3) Endpoints: 30-day testing evaluated repeatedly by household and missed school days
(4) Intercurrent events (ICE): Change of phone number and apathy or inability to report when either asymptomatic and unexposed or symptomatic. In the primary analysis, we take an Intention-to-Treat approach to unobserved outcomes which we assume to be Missing at Random (MAR). In sensitivity analyses, we consider pattern mixture models (under MAR) and plausible outcome conjectures (under a Missing Not at Random assumption - MNAR) to address possible differences in self-reported outcomes by treatment arm.
(5) Population-level summary: relative testing rate and rate of missed school days.

4 ORIGINAL STUDY DESIGN AND MODIFICATIONS:

The trial was designed to be cluster-randomized, enrolling at least 50% of students from each of 26 schools. Type I error would be split evenly (0.025) for each co-primary outcome. The first phase of treatment would be 80% to TM with the anticipation that a sufficient number of TM households would be randomized to TM+HN vs TM with no HN when they declare having symptoms but do not request a test. We assumed a baseline testing rate for TM of 50% and a 15% missing-school rate. Under an intraclass correlation coefficient of 0.02, the trial was 80% powered to detect an increase of 9.2% in testing and a decrease of 6.1% in missed school days.

5 CHANGES TO THE STUDY DESIGN

For logistical reasons, SCALE-UP COUNTS had multiple adaptions to the trial design and estimands of interest. Key stakeholders remained in frequent communication and alignment regarding these changes.

First, because of the low response rates to outcomes assessments and low uptake of HN, the interventions of interest changed to any TM (i.e., from TM or TM+HN) versus usual care (UC). The first-phase allocation ratio changed to 1:1 (from 5:1). Outcomes would still be collected from both arms through text messaging; and, the trial designed a REDCap survey sent to families every month which would ask about testing rates and missed school days. Second, because we were able to make at-home test kits available to all staff, parents/guardians, and

students, and because we decided to provide HN remotely, SCALE-UP COUNTS was able to randomize within schools at the household contact-level with a low risk of contamination. Third, because Salt Lake City School District's enrollment was on an opt-in basis, it was determined to use this district to supplement the Granite School District and charter schools as a descriptive analysis.

6 INTERIM ANALYSES TO DETERMINE AN ADEQUATE SAMPLE SIZE

After 2831 households enrolled, and given a low self-report rate, we revisited our assumptions for power calculations. We masked TM self-reported outcomes to protect from any investigative bias that could come from comparing outcomes in both arms. At this evaluation, households received one or two opportunities to self-report testing. At this point in time, 22% of families self-reported at least once in both the UC and TM arm. Among households with two opportunities to self-report, those who self-reported both instances had a higher testing rate (0.28) than those who self-reported once (0.11). Among households with one opportunity to self-report, the testing rate among self-reporters was 0.23. This provided some insight that the self-report rate may be related to testing rates.

To estimate power, we estimated the testing rate under a pattern mixture model (PMM) framework to predict outcomes when self-reported. We fit a Generalized Estimating Equation model on the UC households adjusting for the number of times an individual was contacted, the individual's proportion of self-reporting, and the calendar date of outcome collection. Calendar date was modeled as a spline with three knots to allow for non-linear trends.

For each TM self-reported outcome, we used the above model to estimate the testing rate and increased the testing rate by Cohen's D for Binomial outcomes. For non-reported TM and UC outcomes, we conservatively assumed there to be no treatment effect. The relative testing rate of non-reported to self-reporters was assumed to range from 0.25 to 1.5.

With the above estimates/assumptions of testing rates, we generated outcomes using a Bernoulli distribution. We then tested for a TM treatment effect using GEE model with a logistic link and adjusting for these same covariates. We bootstrap sampled this process, allowing for increased sample size, and estimated the power as the proportion of times a TM effect was observed. Though our estimand would be further refined and account for changes in enrollment allocation (see below), this helped us determine an appropriate sample size that could be robust to conjectured testing rates when not self-reported.

By the fourth iteration, 1768 UC and 3650 TM households had enrolled. The following power figures were generated

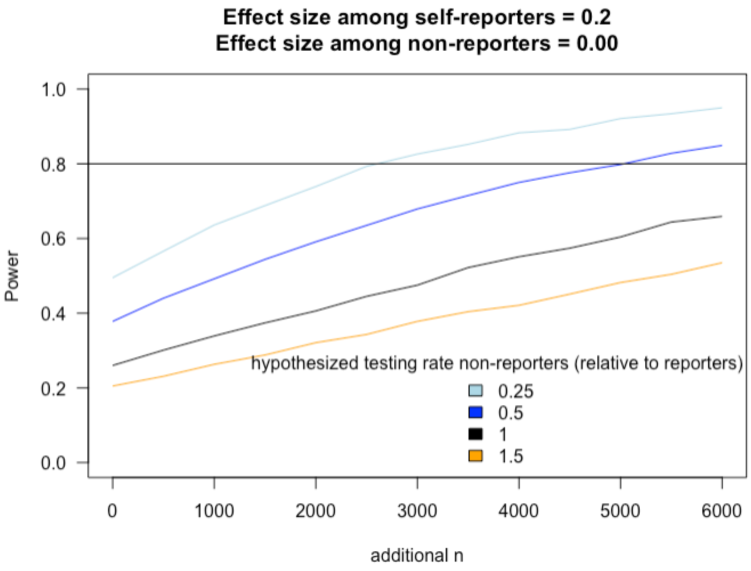

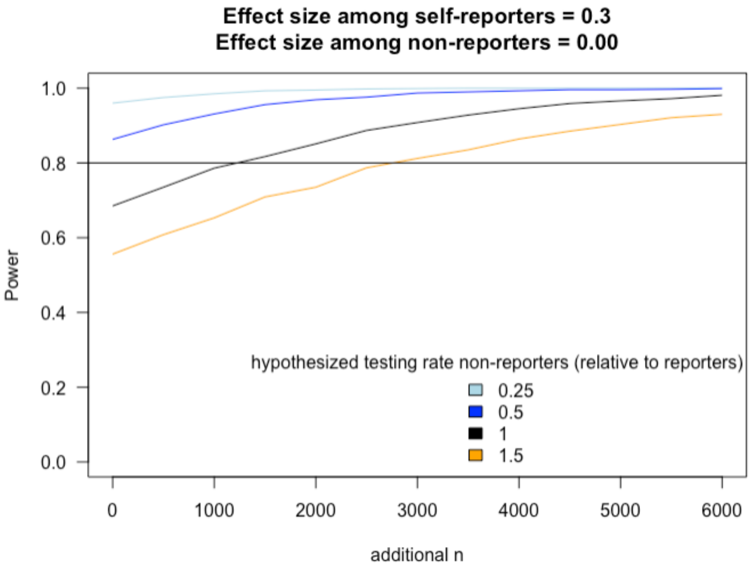

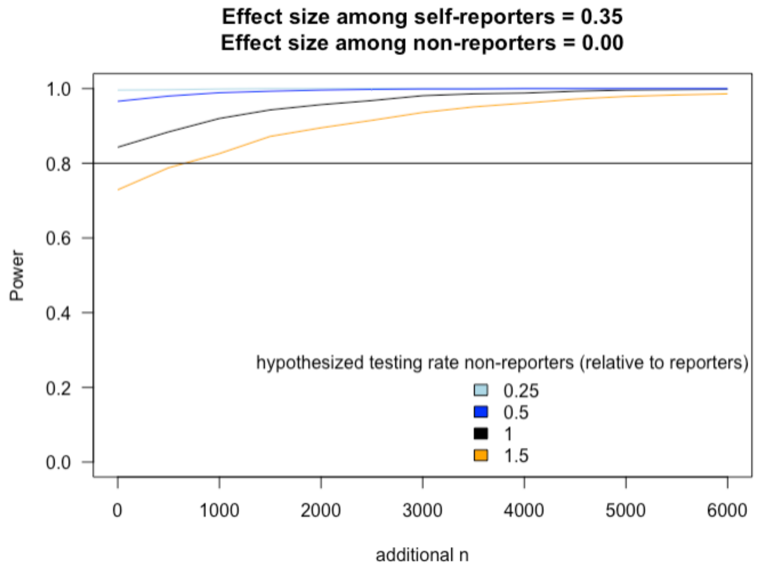

At this point in the study, we aimed to enroll another 1500 households to be 80% powered to detect a TM effect under the following assumptions: a 0.3 effect size among self-reported outcomes (moderately-small), no effect size among non-reporters (conservative), and a similar testing rate among non-reported outcomes (conservative).

7 NEW ESTIMAND (TM VS UC):

With the above modifications, the trial objective became to test whether TM versus UC would differentially (a) Increase the proportion of students tested out of those who meet the criteria for testing and (b) Decrease missed school days. The former would be considered the primary endpoint with the latter a secondary endpoint; both to be tested at alpha of 0.05. The five key elements of the estimand included:

(1) Population: Families with students in the Granite School District and participating charter schools
(2) Treatment: Receipt of TM versus usual care
(3) Endpoints: 30-day testing evaluated repeatedly by household (primary) and missed school days (secondary)
(4) Intercurrent events (ICE): Change of phone number and apathy or inability to report when either asymptomatic and unexposed or symptomatic. In the primary analysis, we take an Intention-to-Treat approach to unobserved outcomes which we assume to be Missing at Random (MAR). In sensitivity

analyses, we consider pattern mixture models (under MAR) and plausible outcome conjectures (under a Missing Not at Random assumption - MNAR) to address possible differences in self-reported outcomes by treatment arm.

(5) Population-level summary: relative testing rate (RTR) and rate of missed school days.

Each household contact, indexed as $i = 1, \dots, n$, received treatment $Z_i = \{0 = UC, 1 = TM\}$ and was assessed a variable number of follow-up times $j = 1, \dots, M_i$ times. Respectively, n_{UC} and n_{TM} indvduals received UC and TM. Households were enrolled in one of two allocation eras $A_i = \{0 = 80\%$ to $TM, 1 = 50\%$ to $TM\}$. In each assessment, the household contact was asked whether any household member tested for COVID positivity in the past 30 days; i.e., the potential outcome $Y_{ij}(Z) = \{0 = no, 1 = yes\}$ denotes any 30-day testing within household i during their j^{th} assessment under treatment Z. Because the prevalence of COVID changed over time, it is anticipated that testing will depend upon an assessment's calendar time, C_{ij}. The summary of interest is the population-level RTR over calendar time in which all calendar time is of equal interest (i.e, $f(C)$ is uniformly distributed over all calendar time of data collection irrespective of treatment arm).

$$RTR = \frac{\int_C E[Y_{ij} \mid Z_i = 1, C_{ij}] f(C) dC}{\int_C E[Y_{ij} \mid Z_i = 0, C_{ij}] f(C) dC}$$

Because the trial enrolled under two eras of different allocation, we will estimate the RTR stratified by the allocation era and pooled by the inverse variance of the estimates. The motivation for this strategy is detailed in the next section.

For the missed school days outcome, $Y_{ij}(Z)$ denotes the number of missed school days in the past 30 days for the first household's first enrolled child. This child's outcomes were chosen because it was the most likely to have data (being listed first in surveys) and because it reflects the family's first exposure the study. The quantity of interest is the Relative Missed Days in past 30 days (RMD30) and is similar to the RTR:

$$RMD30 = \frac{\int_C E[Y_{ij} \mid Z_i = 1, C_{ij}] f(C) dC}{\int_C E[Y_{ij} \mid Z_i = 0, C_{ij}] f(C) dC}$$

8 ENROLLMENT ALLOCATION ERAS

Prior to 7/20/2022, patients in the study were enrolled with a 5:1 allocation ratio favoring TM. After 7/20/22, the allocation ratio changed to 1:1. As a consequence, there is a risk that patients are not equally balanced between arms on key covariates. Compared to UC, TM patients were more heavily enrolled in the earlier allocation period. If a time trend were to have a minimal impact on the outcome, this difference in enrollment times by arm could be inconsequential. However, the COUNTS study added twice as many new schools after the allocation change. Patients in the TM arm are enrolled to a greater extent in the earlier schools than in the UC arm, and any observed treatment differences could be confounded by differences attributable to schools.

Schools enrolled prior to July 2022 allocation change:

(1) Calvin Smith Elementary
(2) Rolling Meadows Elementary
(3) Taylorsville Elementary
(4) Morningside Elementary
(5) Olympus Sr. High
(6) Pioneer Elementary

Schools enrolled after allocation change:

(1) Winter Sports School
(2) Silver Hills Elementary
(3) Hillsdale Elementary
(4) Monroe Elementary
(5) Western Hills Elementary
(6) Utah County Academy of Sciences (UCAS, two locations Orem and Provo)
(7) Evergreen Junior High
(8) Bonneville Junior High
(9) West Kearns El
(10) Oakridge El

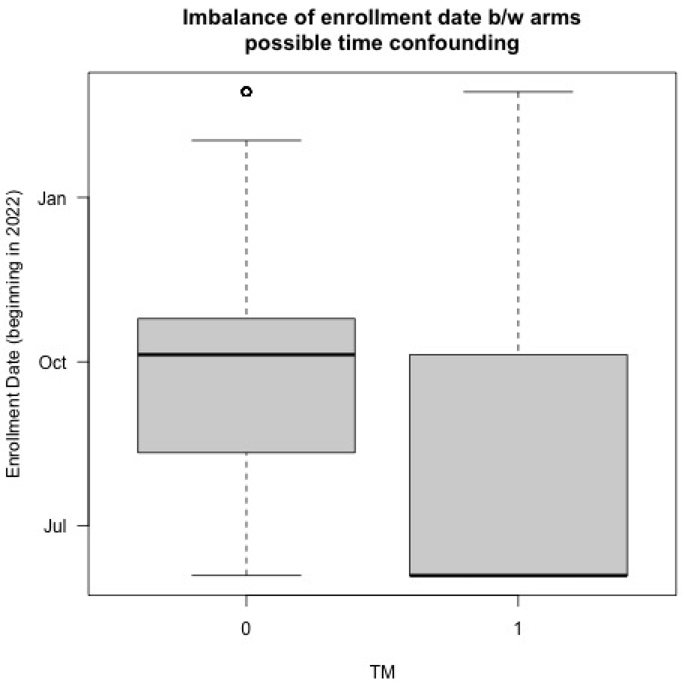

In our analysis, we are adjusting for follow-up calendar time. We looked at the average enrollment date among follow-up assessments across calendar time; however, this did not give reassurance of improved balance between arms.

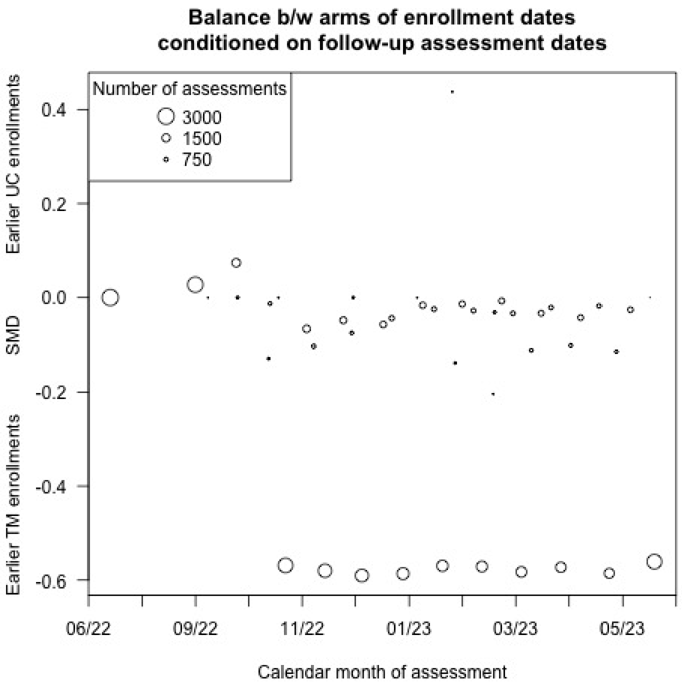

These investigations suggest that the allocation period could be a meaningful confounder, especially given that roughly 2/3rds of schools were enrolled during the second allocation period. Hence, we will stratify by allocation period and pool RTR estimates using inverse-variance weighting.

9 ESTIMATION AND ANALYSIS PLAN FOR TESTING OUTCOME (RTR)

9.1 Primary analysis - observed observations and Missing At Random (MAR) assumption

For the primary analysis, we will perform an Intention to Treat analysis and assume unobserved outcomes are Missing At Random (MAR). The MAR assumption is considered a reasonable starting point for a primary analysis, though sensitivity analyses are recommended because it is typically impossible to guarantee that observed baseline characteristics can sufficiently impute the missing data without bias. Under the MAR assumption, an observed outcomes analysis is approximately unbiased which we will use for the primary analysis. In specifying the mean model, $RCS(C, 5)$ denotes restricted cubic splines with 5 knots placed at the 0.05, 0.275, 0.50, 0.725, and 0.95 quantiles of calendar time, and underlined coefficients correspond with a vector of coefficients:

$$E[Y_{ij} \mid Z_i, C_{ij}, A_i] = \text{logit}^{-1}\left(\beta_0 + \beta_1 \cdot Z_i + \underline{\beta}_2 \cdot RCS(C_{ij}, 5) + \underline{\beta}_3 \cdot RCS(C_{ij}, 5) \cdot Z_i\right).$$

Note that the mean model will be estimated for each allocation period and that the mean model specification allows a separate functional form of testing across calendar time by treatment arm (via the treatment by calendar time interaction). Among observed outcomes, the mean model will be estimated using Generalized Estimating Equations (GEE) with a logit link and a compound symmetry working model for the covariance of household repeated measures. Compound symmetry assumes that testing is equally correlated across a household contact's observations, and this may not be appropriate. For example, a household contact may test positive for COVID then not test for a period of time because an assumed immunity following COVID exposure. We will use robust standard errors to asymptotically correct for misspecification of the working model for covariance. We will use `rcs` function of the `rms` package to create the restricted cubic splines for assessment calendar time. In fitting restricted cubic splines, we may use additional or fewer spline knots if the fit is better and the conclusions are the same.

In estimating the mean model, irregularities could occur because of the complexity allowing a different function form of testing rate over time (i.e., the time by treatment interaction). If this happens, we will simplify the model by removing the calendar time by treatment interaction. This would assume a proportional estimate of testing over time for both treatment groups.

Following GEE estimation, the estimand for each allocation era will be estimated as

$$\widehat{RTR}_{A_i} = \frac{\int_C \hat{E}[Y_{ij} \mid Z_i = 1, C_{ij}, A_i] f(C \mid A_i) dC}{\int_C \hat{E}[Y_{ij} \mid Z_i = 0, C_{ij}, A_i] f(C \mid A_i) dC}.$$

We will follow [1] to combine relative risk estimates across studies accounting for uncertainty. This entails estimating the log-relative risk and standard error (which we will do using bootstrap sampling at the household-level) and aggregating the estimates across allocation eras using inverse variance weighting. A Wald Test will be performed on the log relative risk, and estimates of the mean and confidence interval will be back-transformed to the relative risk scale.

We will also present the estimated probability of testing over time for each treatment group.

9.2 Sensitivity Analysis 1 – Pattern Mixture Model adjusting with MAR assumption

As a first sensitivity analysis, we will additionally adjust for baseline and non-baseline factors that may be associated with testing and response rates. Baseline factors include parent type ($PT_i = \{0 = mother, 1 = father, 2 = other\}$), number of enrolled children (EC_i), and social vulnerability index (SVI_i). Non-baseline factors include whether the household opted out after five assessments ($OO5_i = \{0 = no, 1 = yes\}$) and the household contact's observed self-report rate (SR_i = proportion of self-reported outcomes). For each allocation period, we will fit a separate model for each treatment arm with the mean model specified as:

$$E[Y_{ij} \mid C_{ij}, A_i, PT_i, EC_i, SVI_i, OO5_i, SR_i] =$$
$$\text{logit}^{-1}\left(\beta_0 + \underline{\beta}_1 \cdot RCS(C_{ij}, 5) + \underline{\beta}_2 \cdot PT_i + \beta_3 \cdot EC_i + \underline{\beta}_4 \cdot RCS(SVI_i, 5) + \beta_5 \cdot OO5_i + \beta_6 \cdot SR_i + \beta_7 \cdot OO5_i \cdot SR_i\right).$$

Among observed observations, the mean model will be estimated using GEE with a compound symmetry working model for covariance and robust standard errors. The same limitations regarding mispecifying the working covariance matrix apply.

This is a pattern mixture model [3], and it posits that each individual has a probability of testing, over time, that is fully predictable given the adjusting covariates. A limitation of SR_i is that it may not be well-estimated with relatively few assessments. There may be notable noise in estimating an individual's true SR_i.

For each household contact, we will estimate the testing rate over time given their baseline and post-baseline factors. Although individuals enroll at different times, we will estimate their testing rate throughout the study time; study time is specific to the enrollment allocation era. To integrate out the post-baseline factors, we will average across the household contact-specific functional form of testing rate over time. (This also effectively integrates out over baseline covariates) A household contact, i, which opted out after randomization and before their first assessment will be included with $OO5_i = 0$ and $SR_i = 0$.

The RTR will be estimated as

$$\widehat{RTR}_{A_i} = \frac{\int_C \int_{OO5} \int_{SR} \widehat{E}[Y_{ij} \mid Z_i = 1, C_{ij}, A_i, PT_i, EC_i, SVI_i, OO5_i, SR_i] f(C \mid A_i) f(OO5_i, SR_i \mid Z_i = 1) d(OO5, SR) dC}{\int_C \int_{OO5} \int_{SR} \widehat{E}[Y_{ij} \mid Z_i = 0, C_{ij}, A_i, PT_i, EC_i, SVI_i, OO5_i, SR_i] f(C \mid A_i) f(OO5_i, SR_i \mid Z_i = 0) d(OO5, SR) dC}$$

$$\approx \frac{\int_C \left(\frac{1}{n_{TM}} \sum_{i \in TM} \widehat{E}[Y_{ij} \mid Z_i = 1, C_{ij}, A_i, PT_i, EC_i, SVI_i, OO5_i, SR_i] \right) f(C \mid A_i) d(OO5, SR) dC}{\int_C \left(\frac{1}{n_{UC}} \sum_{i \in UC} \widehat{E}[Y_{ij} \mid Z_i = 0, C_{ij}, A_i, PT_i, EC_i, SVI_i, OO5_i, SR_i] \right) f(C \mid A_i) d(OO5, SR) dC}$$

The overall RTR will be aggregated across allocation eras as in the primary analysis. The utility of this sensitivity analysis depends upon the extent that the adjustment covariates predict well the outcomes and the missingness mechanism. As a limitation, the model conditions on the household contact's observed self-report rate which could be a poor estimation of the household contact's true response rate. This latter concern is magnified when there are few assessments.

Based upon initial assessment, we anticipate less than 1% of individuals have a missing SVI or number of enrolled children. Given the sparse extent of missing data, we will use mean imputation conditioned on school enrollment.

In the primary analysis, we may consider additional knots for the splines on continuous covariates if the model feasibly allows (without convergence issues) and the conclusions are consistent. We will take the same approach in the sensitivity analysis. However, we will aim to use the same number of knots between the analyses if feasible and if conclusions are robust.

9.3 Sensitivity Analysis 2 – Conjecture of testing rates when unreported, missing not at random assumption (MNAR)

With a large extent of unreported outcomes, the MAR assumption is questionable. To relax this assumption, we make a set of conjectures of the testing rate among the unreported outcomes. These are not verifiable conjectures; however, they can serve to place a set of bounds of effects sizes under plausible and stringent assumptions.

In sensitivity analysis 1, we assumed the unobserved outcomes were predictable based upon the observed observations and accounting for post-randomization factors. In this analysis, we still draw upon post-randomization

factors but assume the testing rate is only a fraction of the predicted amount. As in sensitivity analysis 1, we will again estimate each household contact's testing rate over time and average across the household contact testing rates so to integrate out the post-randomization features.

Denote whether an individual responds as R_{ij}. For a household contact in the TM arm, their expected testing rate over time will be modeled as:

$$E[Y_{ij} \mid Z_i = 1, C_{ij}, A_i, PT_i, EC_i, SVI_i, OO5_i, SR_i] \cdot 1(R_{ij} = 1) +$$
$$\gamma_1 E[Y_{ij} \mid Z_i = 1, C_{ij}, A_i, PT_i, EC_i, SVI_i, OO5_i, SR_i] \cdot 1(R_{ij} = 0).$$

Note that $E[Y_{ij} \mid Z_i = 1, C_{ij}, A_i, PT_i, EC_i, SVI_i, OO5_i, SR_i]$ is the model for the TM arm in sensitivity analysis 1. Similarly, for a household contact in the UC arm, their expected testing rate over time will be modeled as:

$$E[Y_{ij} \mid Z_i = 0, C_{ij}, A_i, PT_i, EC_i, SVI_i, OO5_i, SR_i] \cdot 1(SR_{ij} = 1) +$$
$$\gamma_0 E[Y_{ij} \mid Z_i = 0, C_{ij}, A_i, PT_i, EC_i, SVI_i, OO5_i, SR_i] \cdot 1(SR_{ij} = 0).$$

We will consider multiple combinations of γ_1 and γ_0 such as keeping them the same and ranging from 0 to 1.25 by 0.25. When $\gamma_1 = \gamma_0 = 0$, is a meaningful boundary in which a non-reported outcome corresponds with no testing. And, $\gamma_1 = \gamma_0 = 1$ corresponds with sensitivity analysis 1. We will also consider fixing γ_1 to be between 0 and 1.25 and setting γ_0 such that γ_1/γ_0 is proportional by $\alpha\ \widehat{RTR}$ from the primary analysis.

Given a household contact's new testing rate over time, we will mimic sensitivity analysis 1 in which we integrate across household contacts to estimate the testing rate across groups and then integrate over calendar time to estimate the RTR. We will aggregate results across allocation eras as in the primary and sensitivity 1 analysis. We will report the combination of γ_1 and γ_0 values which would nullify the effect of TM.

10 ESTIMATION AND ANALYSIS PLAN FOR MISSED SCHOOL DAYS OUTCOME (RMD30)

The analysis for missed school days will mirror the testing outcome analysis. The quantity of interest is the RMD30 relative number of missed school days (over the past 30 days).

The mean model for the primary and sensitivity analyses will be specified as in the testing outcome model, but with specifying a poison model with a log link. If the model does not converge, we will use a normal model with an identity link and rely upon asymptotic normality. Uncertainty will be modeled and captured as in the estimation of the RTR and results will be similarly pooled.

11 TESTING FOR AN SVI EFFECT MODIFICATION AND ESTIMATION WITHIN SVI LEVELS

To test SVI as a potential effect modifier, we will dichotomize SVI at 0.50 and evaluate the difference in the log RTR (and log RMD30) when SVI is low versus when it is high. Now, we define $SVI{-}LH_i \in \{0 = \text{SVI under } 50, 1 = \text{SVI } 50+\}$.

SVI is ranked based, hence it motivates using 0.5 as a scientifically meaningful threshold (above versus below median SVI). We considered evaluating for an SVI effect modification by using splines on SVI. However, RTR and RMD30 already have the complexities of using splines on calendar time to estimate the treatment effect over time and by arm. For this reason, and given a low self-report rate, we opted for the simplicity of dichotomizing SVI at 0.50.

In accordance with the primary analysis (i.e., using observed outcomes), we will estimate and test the effect modification upon RTR by first fitting the following models within each respective treatment arm:

$$E[Y_{ij} \mid Z_i = 0, C_{ij}, A_i, SVI - LH] = \text{logit}^{-1}\left(\beta_0 + \beta_1 \cdot SVI - LH_i + \underline{\beta}_2 \cdot RCS(C_{ij}, 5) + \underline{\beta}_3 \cdot RCS(C_{ij}, 5) \cdot SVI_i\right).$$
$$E[Y_{ij} \mid Z_i = 1, C_{ij}, A_i, SVI - LH] = \text{logit}^{-1}\left(\beta_0 + \beta_1 \cdot SVI - LH_i + \underline{\beta}_2 \cdot RCS(C_{ij}, 5) + \underline{\beta}_3 \cdot RCS(C_{ij}, 5) \cdot SVI_i\right).$$

The RTR for a given allocation era and SVI level is then:

$$\widehat{RTR}_{A_i|SVI-LH=Low} = \frac{\int_C \hat{E}[Y_{ij} \mid Z_i = 1, C_{ij}, A_i, SVI - LH = Low] f(C \mid A_i) dC}{\int_C \hat{E}[Y_{ij} \mid Z_i = 0, C_{ij}, A_i, SVI - LH = Low] f(C \mid A_i) dC}, \text{ and}$$
$$\widehat{RTR}_{A_i|SVI-LH=Hi} = \frac{\int_C \hat{E}[Y_{ij} \mid Z_i = 1, C_{ij}, A_i, SVI - LH = Hi] f(C \mid A_i) dC}{\int_C \hat{E}[Y_{ij} \mid Z_i = 0, C_{ij}, A_i, SVI - LH = Hi] f(C \mid A_i) dC}.$$

Then, on the log-scale, we will estimate the possible differential RTR effect when SVI is low versus when SVI is high:

$$\text{SVI effect modification} = log(RTR_{A_i|SVI-LH=Low}) - log(RTR_{A_i|SVI-LH=Hi})$$

We will bootstrap sample to obtain an estimate of the effect modification. This will be done separately for each allocation era, and the overall estimate will be pooled and tested as in the primary analysis. Regardless of statistical significance, we will provide estimates of the RTR within each SVI level aggregated across allocation eras by estimating the log RTR, estimating the variance through bootstrap sampling, hypothesis testing of the log RTR, and back-transforming to the RTR scale.

We will follow the same analytic strategy to test for an effect modification upon RMD30 and to estimate the treatment effect within SVI subgroups.

We will further test for effect modification under the PMM model. In this sensitivity analysis, we will modify the outcome model for each arm as:

$$E[Y_{ij} \mid C_{ij}, A_i, PT_i, EC_i, SVI_i, OO5_i, SR_i] =$$
$$\text{logit}^{-1}(\beta_0 + \underline{\beta}_1 \cdot SVI - LH_i + \underline{\beta}_2 \cdot RCS(C_{ij}, 5) + \underline{\beta}_3 \cdot SVI - LH_i \cdot RCS(C_{ij}, 5) +$$
$$\underline{\beta}_4 \cdot PT_i + \beta_5 \cdot EC_i + \underline{\beta}_6 \cdot OO5_i + \beta_7 \cdot SR_i + \beta_8 \cdot OO5_i \cdot SR_i).$$

Here, the change is to dichotomize SVI to SVI-LH and to include an SVI and calendar time interaction.

With this modification, estimation and testing for testing and missed school days outcomes proceeds as in the observed outcomes analysis.

REFERENCES

[1] Friedrich, J. O., Adhikari, N. K. J., and Beyene, J. (2008). The ratio of means method as an alternative to mean differences for analyzing continuous outcome variables in meta-analysis: a simulation study. *BMC Medical Research Methodology*, 8:1–15.

[2] International Council for Harmonisation of Technical Requirements for Pharmaceuticals for Human use (2017). ICH Harmonized Guideline E9 (R1) Estimands and Sensitivity analysis in Clinical Trials.

[3] Little, R. J. A. (1993). Pattern-mixture models for multivariate incomplete data. *Journal of the American Statistical Association*, 88(421):125–134.
