# Supplementary material for: Mobile Intervention for Increasing COVID-19 Testing in K-12 Schools Serving Disadvantaged Communities: Randomized Controlled Trial of SCALE-UP Counts
Source: J Med Internet Res. 2025 Nov 11;27:e79775. doi: 10.2196/79775 (PMC12648121; doi:10.2196/79775)
Supplement: Multimedia Appendix 2 [file jmir_v27i1e79775_app2.pdf]

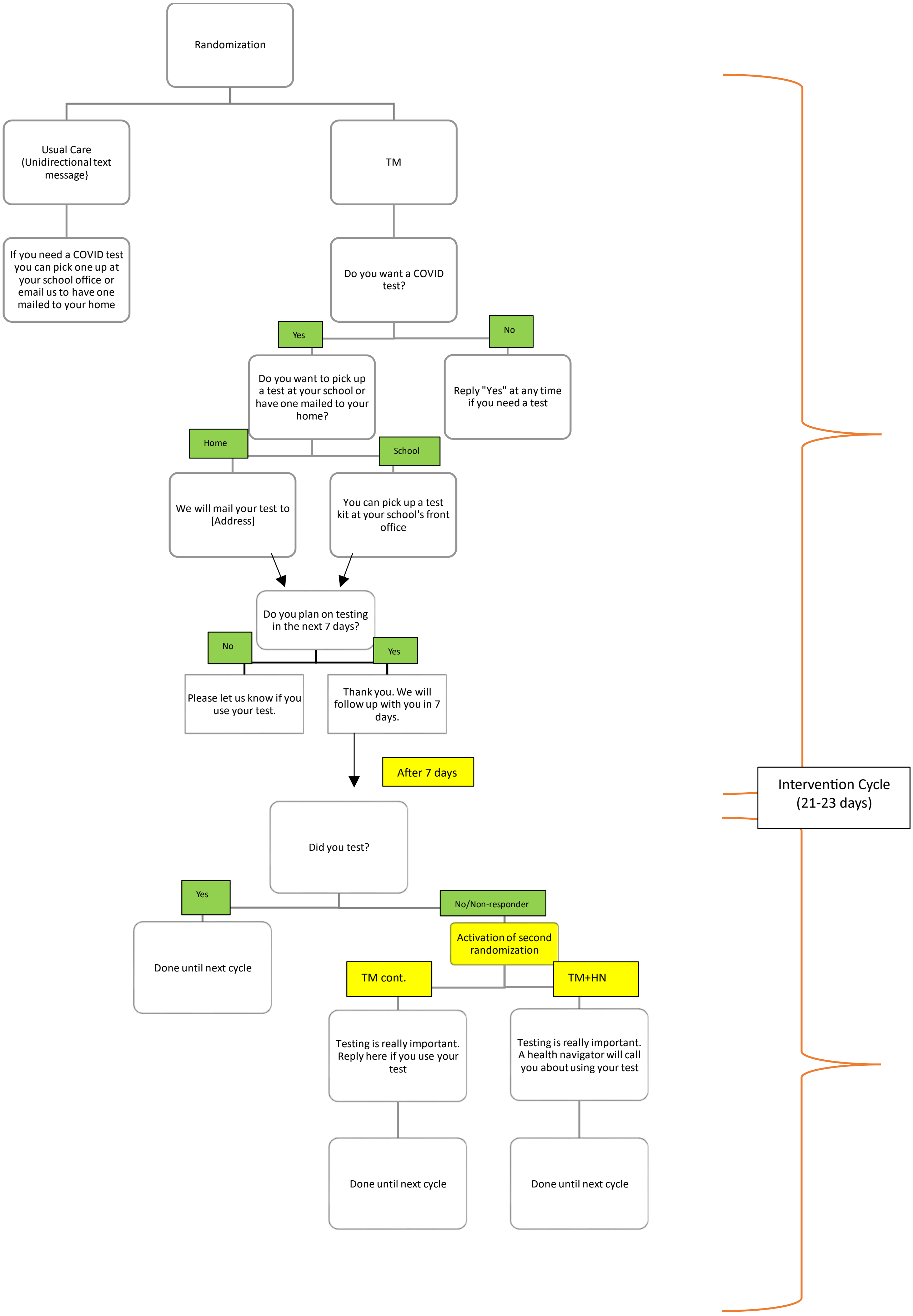

Randomization
Usual Care (Unidirectional text message}
TM
If you need a COVID test you can pick one up at your school office or email us to have one mailed to your home
Do you want a COVID test?
Yes
No
Do you want to pick up a test at your school or have one mailed to your home?
Reply "Yes" at any time if you need a test
Home
School
We will mail your test to [Address]
You can pick up a test kit at your school's front office
Do you plan on testing in the next 7 days?
No
Yes
Please let us know if you use your test.
Thank you. We will follow up with you in 7 days.
After 7 days
Did you test?
Yes
No/Non-responder
Done until next cycle
Activation of second randomization
TM cont.
TM+HN
Testing is really important. Reply here if you use your test
Testing is really important. A health navigator will call you about using your test
Done until next cycle
Done until next cycle
Intervention Cycle (21-23 days)

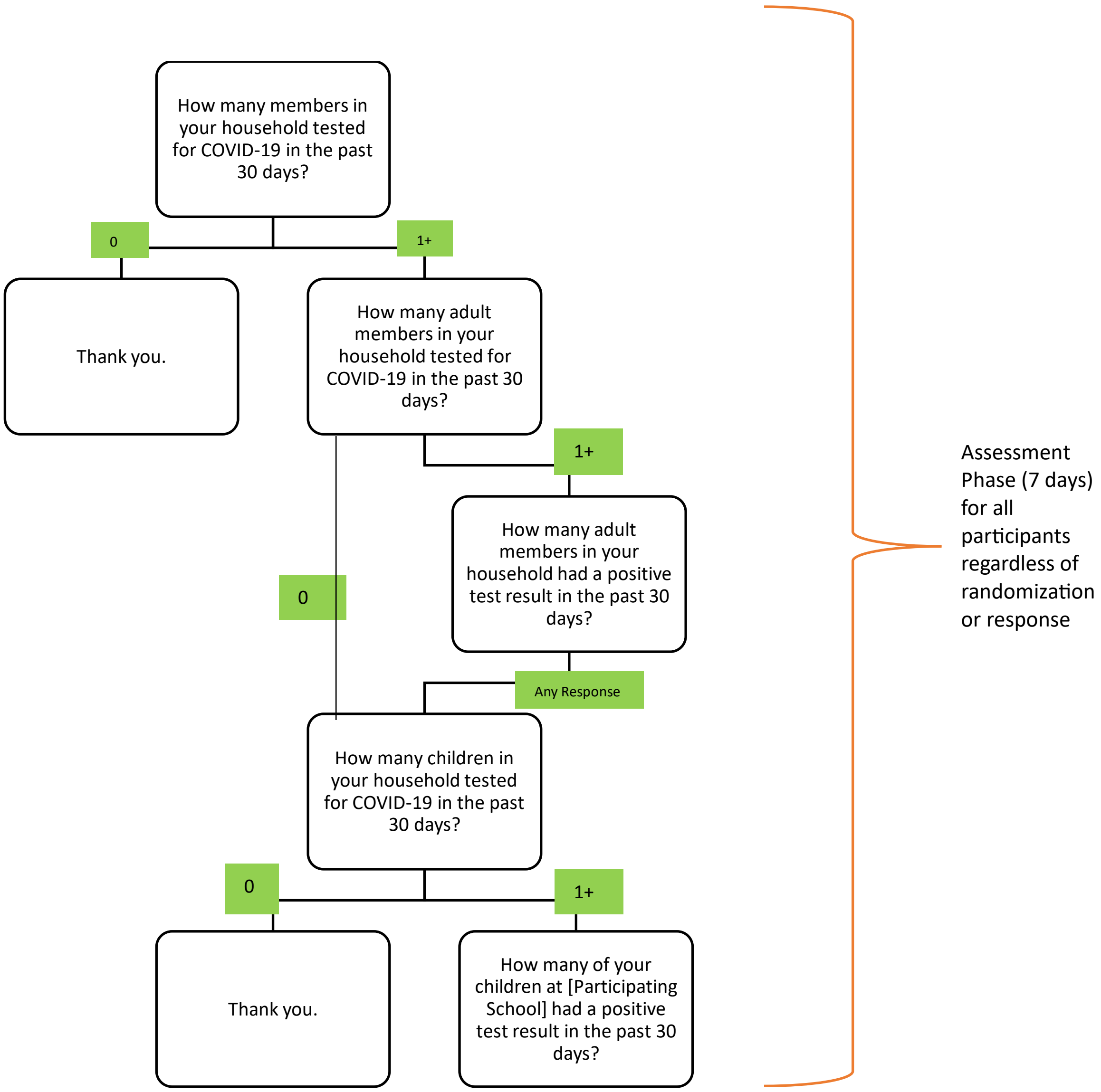

Note. This figure displays the study's longest running text message campaign (May 2022-June 2023). At the start of the study (Feb 2022-May 2022), the initial text messaging campaign assessed for COVID-19 symptoms and exposures.
