# Supplementary material for: Mobile Intervention for Increasing COVID-19 Testing in K-12 Schools Serving Disadvantaged Communities: Randomized Controlled Trial of SCALE-UP Counts
Source: J Med Internet Res. 2025 Nov 11;27:e79775. doi: 10.2196/79775 (PMC12648121; doi:10.2196/79775)
Supplement: Multimedia Appendix 4 [file jmir_v27i1e79775_app4.docx]

**Table S1.** Relative Testing Rate (RTR) of intensive text messaging (TM) versus usual care or control (UC) and sensitivity analyses by allocation era; effect within Social Vulnerability Index (SVI) levels.

| Allocation: 80% TM, 20% UC | | | | |
| --- | --- | --- | --- | --- |
|  | RTR | Lower 95% | Upper 95% | p-value |
| Observed Outcomes: |  |  |  |  |
| SVI < 50 | 1.43 | 1.19 | 1.72 | < 0.001 |
| SVI 50+ | 1.64 | 1.23 | 2.18 | 0.001 |
| Pattern Mixture Model: |  |  |  |  |
| SVI < 50 | 1.48 | 1.04 | 2.09 | 0.028 |
| SVI 50+ | 1.64 | 1.11 | 2.42 | 0.012 |

| Allocation: 50% TM, 50% UC | | | | |
| --- | --- | --- | --- | --- |
|  | RTR | Lower 95% | Upper 95% | p-value |
| Observed Outcomes: |  |  |  |  |
| SVI < 50 | 1.40 | 1.15 | 1.72 | 0.001 |
| SVI 50+ | 1.62 | 1.32 | 1.98 | < 0.001 |
| Pattern Mixture Model: |  |  |  |  |
| SVI < 50 | 1.67 | 1.12 | 2.47 | 0.011 |
| SVI 50+ | 1.78 | 1.30 | 2.44 | < 0.001 |

**Table S2.** Relative Missed School Days Rate (RMD 30) of intensive text messaging (TM) versus usual care or control (UC) and sensitivity analyses by allocation era; effect within Social Vulnerability Index (SVI) levels.

| Allocation: 80% TM, 20% UC | | | | |
| --- | --- | --- | --- | --- |
|  | RMD30 | Lower 95% | Upper 95% | p-value |
| Observed Outcomes: | 1.15 | 0.51 | 2.62 | 0.73 |
| SVI < 50 | 0.98 | 0.26 | 3.73 | 0.98 |
| SVI 50+ | 1.40 | 0.33 | 5.92 | 0.65 |
| Pattern Mixture Model: | 0.66 | 0.19 | 2.31 | 0.52 |
| SVI < 50 | 0.64 | 0.21 | 1.98 | 0.44 |
| SVI 50+ | 0.69 | 0.15 | 3.15 | 0.63 |

| Allocation: 50% TM, 50% UC | | | | |
| --- | --- | --- | --- | --- |
|  | RMD30 | Lower 95% | Upper 95% | p-value |
| Observed Outcomes: | 1.77 | 1.02 | 3.07 | 0.044 |
| SVI < 50 | 1.75 | 0.93 | 3.30 | 0.082 |
| SVI 50+ | 1.79 | 0.87 | 3.71 | 0.12 |
| Pattern Mixture Model: | 1.57 | 0.73 | 3.39 | 0.25 |
| SVI < 50 | 1.46 | 0.64 | 3.36 | 0.37 |
| SVI 50+ | 1.62 | 0.73 | 3.60 | 0.24 |
