# Supplementary material for: Mobile Intervention for Increasing COVID-19 Testing in K-12 Schools Serving Disadvantaged Communities: Randomized Controlled Trial of SCALE-UP Counts
Source: J Med Internet Res. 2025 Nov 11;27:e79775. doi: 10.2196/79775 (PMC12648121; doi:10.2196/79775)
Supplement: Multimedia Appendix 3 [file jmir_v27i1e79775_app3.pdf]

CONSORT-EHEALTH (V 1.6.1) - Submission/Publication Form

The CONSORT-EHEALTH checklist is intended for authors of randomized trials evaluating web-based and Internet-based applications/interventions, including mobile interventions, electronic games (incl multiplayer games), social media, certain telehealth applications, and other interactive and/or networked electronic applications. Some of the items (e.g. all subitems under item 5 - description of the intervention) may also be applicable for other study designs.

The goal of the CONSORT EHEALTH checklist and guideline is to be
a) a guide for reporting for authors of RCTs,
b) to form a basis for appraisal of an ehealth trial (in terms of validity)

CONSORT-EHEALTH items/subitems are MANDATORY reporting items for studies published in the Journal of Medical Internet Research and other journals / scientific societies endorsing the checklist.

Items numbered 1., 2., 3., 4a., 4b etc are original CONSORT or CONSORT-NPT (non-pharmacologic treatment) items.
Items with Roman numerals (i., ii, iii, iv etc.) are CONSORT-EHEALTH extensions/clarifications.

As the CONSORT-EHEALTH checklist is still considered in a formative stage, we would ask that you also RATE ON A SCALE OF 1-5 how important/useful you feel each item is FOR THE PURPOSE OF THE CHECKLIST and reporting guideline (optional).

Mandatory reporting items are marked with a red *.
In the textboxes, either copy & paste the relevant sections from your manuscript into this form - please include any quotes from your manuscript in QUOTATION MARKS,
or answer directly by providing additional information not in the manuscript, or elaborating on why the item was not relevant for this study.

YOUR ANSWERS WILL BE PUBLISHED AS A SUPPLEMENTARY FILE TO YOUR PUBLICATION IN JMIR AND ARE CONSIDERED PART OF YOUR PUBLICATION (IF ACCEPTED).
Please fill in these questions diligently. Information will not be copyedited, so please use proper spelling and grammar, use correct capitalization, and avoid abbreviations.

DO NOT FORGET TO SAVE AS PDF _AND_ CLICK THE SUBMIT BUTTON SO YOUR ANSWERS ARE IN OUR DATABASE !!!

Citation Suggestion (if you append the pdf as Appendix we suggest to cite this paper in the caption):
Eysenbach G, CONSORT-EHEALTH Group
CONSORT-EHEALTH: Improving and Standardizing Evaluation Reports of Web-based and Mobile Health Interventions
J Med Internet Res 2011;13(4):e126
URL: http://www.jmir.org/2011/4/e126/
doi: 10.2196/jmir.1923
PMID: 22209829

courtney.carroll@hsc.utah.edu Switch account
Not shared
Draft saved

* Indicates required question

Your name *
First Last

Yelena Wu

Primary Affiliation (short), City, Country *
University of Toronto, Toronto, Canada

University of Utah, Salt Lake City, USA

Your e-mail address *
abc@gmail.com

Yelena.Wu@hci.utah.edu

Title of your manuscript *
Provide the (draft) title of your manuscript.

Mobile Intervention for Increasing COVID-19 Testing in K-12 Schools Serving Disadvantaged Communities: Randomized Controlled Trial of SCALE-UP Counts

Name of your App/Software/Intervention *
If there is a short and a long/alternate name, write the short name first and add the long name in brackets.

SCALE-UP Counts

Evaluated Version (if any)
e.g. "V1", "Release 2017-03-01", "Version 2.0.27913"

Your answer

Language(s) *
What language is the intervention/app in? If multiple languages are available, separate by comma (e.g. "English, French")

English, Spanish

URL of your Intervention Website or App
e.g. a direct link to the mobile app on app in appstore (itunes, Google Play), or URL of the website. If the intervention is a DVD or hardware, you can also link to an Amazon page.

https://healthcare.utah.edu/huntsmancancerinstitute/clinical-trials/scale-up-counts-study

URL of an image/screenshot (optional)

Your answer

Accessibility *
Can an enduser access the intervention presently?

- [ ] access is free and open
- [ ] access only for special usergroups, not open
- [ ] access is open to everyone, but requires payment/subscription/in-app purchases
- [x] app/intervention no longer accessible
- [ ] Other:

Primary Medical Indication/Disease/Condition *
e.g. "Stress", "Diabetes", or define the target group in brackets after the condition, e.g. "Autism (Parents of children with)", "Alzheimers (Informal Caregivers of)"

COVID-19

Primary Outcomes measured in trial *
comma-separated list of primary outcomes reported in the trial

Proportion of parents whose households teste

Secondary/other outcomes
Are there any other outcomes the intervention is expected to affect?

Average number of monthly school days missed per participating child

Recommended "Dose" *
What do the instructions for users say on how often the app should be used?

- [ ] Approximately Daily
- [ ] Approximately Weekly
- [ ] Approximately Monthly
- [ ] Approximately Yearly
- [x] "as needed"
- [ ] Other:

Approx. Percentage of Users (starters) still using the app as recommended after 3 months *

- [ ] unknown / not evaluated
- [ ] 0-10%
- [ ] 11-20%
- [ ] 21-30%
- [ ] 31-40%
- [ ] 41-50%
- [ ] 51-60%
- [ ] 61-70%
- [ ] 71%-80%
- [ ] 81-90%
- [ ] 91-100%
- [x] Other: Due to the in-depth nature of the use data, we are reporting it in a sepa

Overall, was the app/intervention effective? *

- [x] yes: all primary outcomes were significantly better in intervention group vs control
- [ ] partly: SOME primary outcomes were significantly better in intervention group vs control
- [ ] no statistically significant difference between control and intervention
- [ ] potentially harmful: control was significantly better than intervention in one or more outcomes
- [ ] inconclusive: more research is needed
- [ ] Other:

Article Preparation Status/Stage *
At which stage in your article preparation are you currently (at the time you fill in this form)

- [ ] not submitted yet - in early draft status
- [ ] not submitted yet - in late draft status, just before submission
- [ ] submitted to a journal but not reviewed yet
- [ ] submitted to a journal and after receiving initial reviewer comments
- [x] submitted to a journal and accepted, but not published yet
- [ ] published
- [ ] Other:

Journal *
If you already know where you will submit this paper (or if it is already submitted), please provide the journal name (if it is not JMIR, provide the journal name under "other")

- [ ] not submitted yet / unclear where I will submit this
- [x] Journal of Medical Internet Research (JMIR)
- [ ] JMIR mHealth and UHealth
- [ ] JMIR Serious Games
- [ ] JMIR Mental Health
- [ ] JMIR Public Health
- [ ] JMIR Formative Research
- [ ] Other JMIR sister journal
- [ ] Other:

Is this a full powered effectiveness trial or a pilot/feasibility trial? *

- [ ] Pilot/feasibility
- [x] Fully powered

Manuscript tracking number *
If this is a JMIR submission, please provide the manuscript tracking number under "other" (The ms tracking number can be found in the submission acknowledgement email, or when you login as author in JMIR. If the paper is already published in JMIR, then the ms tracking number is the four-digit number at the end of the DOI, to be found at the bottom of each published article in JMIR)

- [ ] no ms number (yet) / not (yet) submitted to / published in JMIR
- [x] Other: 79775

TITLE AND ABSTRACT

1a) TITLE: Identification as a randomized trial in the title

1a) Does your paper address CONSORT item 1a? *
I.e does the title contain the phrase "Randomized Controlled Trial"? (if not, explain the reason under "other")

- [x] yes
- [ ] Other:

1a-i) Identify the mode of delivery in the title
Identify the mode of delivery. Preferably use “web-based” and/or “mobile” and/or “electronic game” in the title. Avoid ambiguous terms like “online”, “virtual”, “interactive”. Use “Internet-based” only if Intervention includes non-web-based Internet components (e.g. email), use “computer-based” or “electronic” only if offline products are used. Use “virtual” only in the context of “virtual reality” (3-D worlds). Use “online” only in the context of “online support groups”. Complement or substitute product names with broader terms for the class of products (such as “mobile” or “smart phone” instead of “iphone”), especially if the application runs on different platforms.

	1	2	3	4	5	
subitem not at all important	○	○	○	○	○	essential

Does your paper address subitem 1a-i? *
Copy and paste relevant sections from manuscript title (include quotes in quotation marks "like this" to indicate direct quotes from your manuscript), or elaborate on this item by providing additional information not in the ms, or briefly explain why the item is not applicable/relevant for your study

Mobile Intervention for Increasing COVID-19 Testing in K-12 Schools Serving Disadvantaged Communities

1a-ii) Non-web-based components or important co-interventions in title
Mention non-web-based components or important co-interventions in title, if any (e.g., “with telephone support”).

	1	2	3	4	5	
subitem not at all important	○	○	○	○	○	essential

Does your paper address subitem 1a-ii?
Copy and paste relevant sections from manuscript title (include quotes in quotation marks "like this" to indicate direct quotes from your manuscript), or elaborate on this item by providing additional information not in the ms, or briefly explain why the item is not applicable/relevant for your study

Not applicable- there was a co-intervention of adding health navigator phone calls (TM+HN) to a subset of people receiving the TM intervention, but the rate of participants qualifying as non-responders and the overall self-report were too low to meaningfully make comparisons between TM and TM+HN.

1a-iii) Primary condition or target group in the title
Mention primary condition or target group in the title, if any (e.g., “for children with Type I Diabetes”) Example: A Web-based and Mobile Intervention with Telephone Support for Children with Type I Diabetes: Randomized Controlled Trial

	1	2	3	4	5	
subitem not at all important	○	○	○	○	○	essential

Does your paper address subitem 1a-iii? *
Copy and paste relevant sections from manuscript title (include quotes in quotation marks "like this" to indicate direct quotes from your manuscript), or elaborate on this item by providing additional information not in the ms, or briefly explain why the item is not applicable/relevant for your study

Increasing COVID-19 Testing in K-12 Schools Serving Disadvantaged Communities

1b) ABSTRACT: Structured summary of trial design, methods, results, and conclusions
NPT extension: Description of experimental treatment, comparator, care providers, centers, and blinding status.

1b-i) Key features/functionalities/components of the intervention and comparator in the METHODS section of the ABSTRACT
Mention key features/functionalities/components of the intervention and comparator in the abstract. If possible, also mention theories and principles used for designing the site. Keep in mind the needs of systematic reviewers and indexers by including important synonyms. (Note: Only report in the abstract what the main paper is reporting. If this information is missing from the main body of text, consider adding it)

	1	2	3	4	5	
subitem not at all important	○	○	○	○	○	essential

Does your paper address subitem 1b-i? *
Copy and paste relevant sections from the manuscript abstract (include quotes in quotation marks "like this" to indicate direct quotes from your manuscript), or elaborate on this item by providing additional information not in the ms, or briefly explain why the item is not applicable/relevant for your study

Sequential Multiple Assignment Randomized Trial; compared results between 2 unblinded interventions, 1. intensive, fully automated, bidirectional text messaging about COVID-19 testing; or 2. control- fully automated unidirectional text messaging about COVID-19 testing; both arms of trial provided schools free COVID-19 test kits

1b-ii) Level of human involvement in the METHODS section of the ABSTRACT
Clarify the level of human involvement in the abstract, e.g., use phrases like “fully automated” vs. “therapist/nurse/care provider/physician-assisted” (mention number and expertise of providers involved, if any). (Note: Only report in the abstract what the main paper is reporting. If this information is missing from the main body of text, consider adding it)

	1	2	3	4	5	
subitem not at all important	○	○	○	○	○	essential

Does your paper address subitem 1b-ii?
Copy and paste relevant sections from the manuscript abstract (include quotes in quotation marks "like this" to indicate direct quotes from your manuscript), or elaborate on this item by providing additional information not in the ms, or briefly explain why the item is not applicable/relevant for your study

fully automated text messaging
There was a co-intervention of adding health navigator phone calls (TM+HN) to a subset of people receiving the TM intervention, but the rate of participants qualifying as nonresponders and the overall self-report were too low to meaningfully make comparisons between TM and TM+HN, so we did not include this in the abstract.

1b-iii) Open vs. closed, web-based (self-assessment) vs. face-to-face assessments in the METHODS section of the ABSTRACT
Mention how participants were recruited (online vs. offline), e.g., from an open access website or from a clinic or a closed online user group (closed usergroup trial), and clarify if this was a purely web-based trial, or there were face-to-face components (as part of the intervention or for assessment). Clearly say if outcomes were self-assessed through questionnaires (as common in web-based trials). Note: In traditional offline trials, an open trial (open-label trial) is a type of clinical trial in which both the researchers and participants know which treatment is being administered. To avoid confusion, use “blinded” or “unblinded” to indicated the level of blinding instead of “open”, as “open” in web-based trials usually refers to “open access” (i.e. participants can self-enrol). (Note: Only report in the abstract what the main paper is reporting. If this information is missing from the main body of text, consider adding it)

	1	2	3	4	5	
subitem not at all important	○	○	○	○	○	essential

Does your paper address subitem 1b-iii?
Copy and paste relevant sections from the manuscript abstract (include quotes in quotation marks "like this" to indicate direct quotes from your manuscript), or elaborate on this item by providing additional information not in the ms, or briefly explain why the item is not applicable/relevant for your study

Fully automated text messaging; 16 schools; enrolled all eligible participants who did not opt out; asked parents to...self-report measures on testing outcomes and missed school days through web-based questionnaires.

1b-iv) RESULTS section in abstract must contain use data
Report number of participants enrolled/assessed in each group, the use/uptake of the intervention (e.g., attrition/adherence metrics, use over time, number of logins etc.), in addition to primary/secondary outcomes. (Note: Only report in the abstract what the main paper is reporting. If this information is missing from the main body of text, consider adding it)

	1	2	3	4	5	
subitem not at all important	○	○	○	○	○	essential

Does your paper address subitem 1b-iv?
Copy and paste relevant sections from the manuscript abstract (include quotes in quotation marks "like this" to indicate direct quotes from your manuscript), or elaborate on this item by providing additional information not in the ms, or briefly explain why the item is not applicable/relevant for your study

7122 parents of students from 16 schools, half title 1; 2588 randomized to usual care or control and 4534 to bidirectional text messaging; intervention led to increased self-reported testing when compared with the control condition (22.8% vs 13.5%, relative testing rate = 1.64, 95% CI 1.31-2.02; P<.001); no observed difference in missed school days between the study arms
Because information about use is being reported in a separate manuscript that is currently being prepared, we have not provided it in this publication.

1b-v) CONCLUSIONS/DISCUSSION in abstract for negative trials
Conclusions/Discussions in abstract for negative trials: Discuss the primary outcome - if the trial is negative (primary outcome not changed), and the intervention was not used, discuss whether negative results are attributable to lack of uptake and discuss reasons. (Note: Only report in the abstract what the main paper is reporting. If this information is missing from the main body of text, consider adding it)

	1	2	3	4	5	
subitem not at all important	○	○	○	○	○	essential

Does your paper address subitem 1b-v?
Copy and paste relevant sections from the manuscript abstract (include quotes in quotation marks "like this" to indicate direct quotes from your manuscript), or elaborate on this item by providing additional information not in the ms, or briefly explain why the item is not applicable/relevant for your study

"SCALE-UP Counts worked closely with schools and the state’s public health system to implement and test a scalable health information technology approach that delivered automated text messages to students’ parents around COVID-19 testing and provided access to free at-home test kits. Such an approach can help facilitate COVID-19 testing among school communities, including those that provide education and resources to students and their families from racial or ethnic minorities and with low socioeconomic status. Similar health information technology approaches could be used to increase ease of access to testing, reduce testing burden, and provide tailored information on health measures in school communities for a variety of illnesses or public health concerns."

INTRODUCTION

2a) In INTRODUCTION: Scientific background and explanation of rationale

2a-i) Problem and the type of system/solution
Describe the problem and the type of system/solution that is object of the study: intended as stand-alone intervention vs. incorporated in broader health care program? Intended for a particular patient population? Goals of the intervention, e.g., being more cost-effective to other interventions, replace or complement other solutions? (Note: Details about the intervention are provided in “Methods” under 5)

	1	2	3	4	5	
subitem not at all important	○	○	○	○	○	essential

Does your paper address subitem 2a-i? *
Copy and paste relevant sections from the manuscript (include quotes in quotation marks "like this" to indicate direct quotes from your manuscript), or elaborate on this item by providing additional information not in the ms, or briefly explain why the item is not applicable/relevant for your study

"While implementation of school-based and at home testing was associated with increased testing among students, parents, and school staff, several barriers led to lower-than-expected participation rates": knowledge gaps, misinformation, communication barriers, access to testing resources.
Aim: address barriers using stepped approach: "automated bidirectional text messages provided to the school community that alerted parents to the availability of free, rapid COVID-19 tests, provided guidance on when to test, and, for a subgroup of those who needed it, provided access to a health navigator who could address motivations to test, logistical barriers to testing, and questions about COVID-19 testing."
Goals: increase testing; decrease missed school days

2a-ii) Scientific background, rationale: What is known about the (type of) system
Scientific background, rationale: What is known about the (type of) system that is the object of the study (be sure to discuss the use of similar systems for other conditions/diagnoses, if appropiate), motivation for the study, i.e. what are the reasons for and what is the context for this specific study, from which stakeholder viewpoint is the study performed, potential impact of findings [2]. Briefly justify the choice of the comparator.

	1	2	3	4	5	
subitem not at all important	○	○	○	○	○	essential

Does your paper address subitem 2a-ii? *
Copy and paste relevant sections from the manuscript (include quotes in quotation marks "like this" to indicate direct quotes from your manuscript), or elaborate on this item by providing additional information not in the ms, or briefly explain why the item is not applicable/relevant for your study

"Several studies have used similar stepped-care approaches (ie, TM, followed by access to a navigator) to address" health problems "among lower SES, minority, and other vulnerable populations"; "can increase connection to care and improve health outcomes compared with standard care alternatives."
Health navigators "can decrease health disparities by addressing barriers and concerns regarding health care access."
"This study compared these interventions with a control intervention that used unidirectional TM. We selected this control intervention because it controlled for the modality of TM, reflected more typical methods of public health messaging, and allowed participants allocated to the control group to receive relevant COVID-19 testing information."

2b) In INTRODUCTION: Specific objectives or hypotheses

Does your paper address CONSORT subitem 2b? *
Copy and paste relevant sections from the manuscript (include quotes in quotation marks "like this" to indicate direct quotes from your manuscript), or elaborate on this item by providing additional information not in the ms, or briefly explain why the item is not applicable/relevant for your study

"SCALE-UP Counts examined the efficacy of technology-supported interventions in promoting COVID-19 testing among K-12 school communities (students and staff)"; primary outcome: "proportion of parents whose households tested for COVID-19 disease (a relative testing rate [RTR] between study groups)"; secondary outcome: "number of missed school days"; "We hypothesized that the interventions in SCALE-UP Counts, when compared with a control intervention, would increase the proportion of students and school staff who tested for COVID-19 disease and would be related to the number of missed school days."

METHODS

3a) Description of trial design (such as parallel, factorial) including allocation ratio

Does your paper address CONSORT subitem 3a? *
Copy and paste relevant sections from the manuscript (include quotes in quotation marks "like this" to indicate direct quotes from your manuscript), or elaborate on this item by providing additional information not in the ms, or briefly explain why the item is not applicable/relevant for your study

"SCALE-UP Counts was a parallel study designed as a Sequential Multiple Assignment Randomized Trial."; "In the first stage, participants were randomized to intensive TM or usual care or control (UC) using an allocation ratio of 80.0% intensive TM and 20.0% UC"; "second stage of randomization...with an allocation ratio of 50.0% TM and 50.0% TM+HN...The

allocation ratio for the first randomization was updated to 50.0% TM and 50.0% UC after the first year of the study."

3b) Important changes to methods after trial commencement (such as eligibility criteria), with reasons

Does your paper address CONSORT subitem 3b? *

Copy and paste relevant sections from the manuscript (include quotes in quotation marks "like this" to indicate direct quotes from your manuscript), or elaborate on this item by providing additional information not in the ms, or briefly explain why the item is not applicable/relevant for your study

"The allocation ratio for the first randomization was updated to 50.0% TM and 50.0% UC [40] after the first year of the study. This update was made to optimize statistical power to be able to compare intensive TM versus UC intervention arms in end-of-study analyses, because the self-report rate in the first year (20.0%) was much lower than anticipated. This would impact our ability to compare TM with TM+HN. Changes from the original study design and sample size considerations are documented in the statistical analysis plan (Multimedia Appendix 1)."

3b-i) Bug fixes, Downtimes, Content Changes

Bug fixes, Downtimes, Content Changes: ehealth systems are often dynamic systems. A description of changes to methods therefore also includes important changes made on the intervention or comparator during the trial (e.g., major bug fixes or changes in the functionality or content) (5-iii) and other "unexpected events" that may have influenced study design such as staff changes, system failures/downtimes, etc. [2].

1 2 3 4 5

subitem not at all important ○ ○ ○ ○ ○ essential

Does your paper address subitem 3b-i?

Copy and paste relevant sections from the manuscript (include quotes in quotation marks "like this" to indicate direct quotes from your manuscript), or elaborate on this item by providing additional information not in the ms, or briefly explain why the item is not applicable/relevant for your study

"DHARE randomized 61 participants in the UC arm and 119 participants in the TM arm a second time and sent a second initial text message due to a technical error (eg, being listed more than once in the database); we excluded these individuals from analysis."; messaging content "informed by the most recent state and federal public health guidance as well as a pediatric infectious disease specialist. Due to changing COVID-19 testing public health guidance over the course of the study, approximately 2 months into the study, intervention messaging shifted away from COVID-19 testing for diagnostic purposes and toward testing for both diagnostic and preventative purposes."

4a) Eligibility criteria for participants

Does your paper address CONSORT subitem 4a? *

Copy and paste relevant sections from the manuscript (include quotes in quotation marks "like this" to indicate direct quotes from your manuscript), or elaborate on this item by providing additional information not in the ms, or briefly explain why the item is not applicable/relevant for your study

"Parents were eligible to participate if they had a child or student attending a partnering elementary, middle, or high school; had a functioning cellular phone that could receive phone calls and text messages; and had the technology literacy necessary to send text messages."

4a-i) Computer / Internet literacy

Computer / Internet literacy is often an implicit "de facto" eligibility criterion - this should be explicitly clarified.

1 2 3 4 5

subitem not at all important ○ ○ ○ ○ ○ essential

Does your paper address subitem 4a-i?

Copy and paste relevant sections from the manuscript (include quotes in quotation marks "like this" to indicate direct quotes from your manuscript), or elaborate on this item by providing additional information not in the ms, or briefly explain why the item is not applicable/relevant for your study

"Parents were eligible to participate if they...had the technology literacy necessary to send text messages."

4a-ii) Open vs. closed, web-based vs. face-to-face assessments:

Open vs. closed, web-based vs. face-to-face assessments: Mention how participants were recruited (online vs. offline), e.g., from an open access website or from a clinic, and clarify if this was a purely web-based trial, or there were face-to-face components (as part of the intervention or for assessment), i.e., to what degree got the study team to know the participant. In online-only trials, clarify if participants were quasi-anonymous and whether having multiple identities was possible or whether technical or logistical measures (e.g., cookies, email confirmation, phone calls) were used to detect/prevent these.

1 2 3 4 5

subitem not at all important ○ ○ ○ ○ ○ essential

Does your paper address subitem 4a-ii? *

Copy and paste relevant sections from the manuscript (include quotes in quotation marks "like this" to indicate direct quotes from your manuscript), or elaborate on this item by providing additional information not in the ms, or briefly explain why the item is not applicable/relevant for your study

"waiver of documentation of consent"; parents sent initial bidirectional text with study description, link to study website/consent form, option to opt out; follow-up text confirming "they would be receiving text messages regarding COVID-19 testing and could opt out at any time"; fail to opt out within 24 hours = enrolled; schools also distributed flyers in person/social media.
UC: "received unidirectional, static text messages"; testing requested through email;
TM: "sent bidirectional text messages related to COVID-19 testing"; "participant could engage with messaging"; testing requested through text.
TM+HN: Same as TM; each cycle could also include telephone call from health navigator.
Assessments: "assessment phase text message cycles and monthly web-based surveys"

4a-iii) Information giving during recruitment

Information given during recruitment. Specify how participants were briefed for recruitment and in the informed consent procedures (e.g., publish the informed consent documentation as appendix, see also item X26), as this information may have an effect on user self-selection, user expectation and may also bias results.

1 2 3 4 5

subitem not at all important ○ ○ ○ ○ ○ essential

Does your paper address subitem 4a-iii?

Copy and paste relevant sections from the manuscript (include quotes in quotation marks "like this" to indicate direct quotes from your manuscript), or elaborate on this item by providing additional information not in the ms, or briefly explain why the item is not applicable/relevant for your study

IRB waived documentation of consent; parents sent initial bidirectional text with study description, link to study website/consent form, option to opt out; follow-up text confirming "they would be receiving text messages regarding COVID-19 testing and could opt out at any time"; fail to opt out within 24 hours = enrolled

4b) Settings and locations where the data were collected

Does your paper address CONSORT subitem 4b? *

Copy and paste relevant sections from the manuscript (include quotes in quotation marks "like this" to indicate direct quotes from your manuscript), or elaborate on this item by providing additional information not in the ms, or briefly explain why the item is not applicable/relevant for your study

"Participants in this study included parents or legal guardians of students...from 14 schools (elementary, middle, and high schools) within one of the largest school districts in Utah, plus 2 Utah charter schools."
"Assessments were completed through assessment phase text message cycles and monthly web-based surveys"

4b-i) Report if outcomes were (self-)assessed through online questionnaires

Clearly report if outcomes were (self-)assessed through online questionnaires (as common in web-based trials) or otherwise.

1 2 3 4 5

subitem not at all important ○ ○ ○ ○ ○ essential

Does your paper address subitem 4b-i? *

Copy and paste relevant sections from the manuscript (include quotes in quotation marks "like this" to indicate direct quotes from your manuscript), or elaborate on this item by providing additional information not in the ms, or briefly explain why the item is not applicable/relevant for your study

"Parents completed assessments about themselves, their household, and enrolled children. Assessments were completed through assessment phase text message cycles and monthly web-based surveys"

4b-ii) Report how institutional affiliations are displayed

Report how institutional affiliations are displayed to potential participants [on ehealth media], as affiliations with prestigious hospitals or universities may affect volunteer rates, use, and reactions with regards to an intervention.(Not a required item – describe only if this may bias results)

1 2 3 4 5

subitem not at all important ○ ○ ○ ○ ○ essential

Does your paper address subitem 4b-ii?

Copy and paste relevant sections from the manuscript (include quotes in quotation marks "like this" to indicate direct quotes from your manuscript), or elaborate on this item by providing additional information not in the ms, or briefly explain why the item is not applicable/relevant for your study

"parents were sent an initial Health Insurance Portability and Accountability Act–compliant bidirectional text message that described the study and stated its affiliation with the University of Utah, provided a link to the study website and consent form (both of which were branded with the institutional affiliation and included information on all the interventions being tested)"
"Each survey was branded with the institutional affiliation."

5) The interventions for each group with sufficient details to allow replication, including how and when they were actually administered

5-i) Mention names, credential, affiliations of the developers, sponsors, and owners

Mention names, credential, affiliations of the developers, sponsors, and owners [6] (if authors/evaluators are owners or developer of the software, this needs to be declared in a "Conflict of interest" section or mentioned elsewhere in the manuscript).

1 2 3 4 5

subitem not at all important ○ ○ ○ ○ ○ essential

Does your paper address subitem 5-i?

Copy and paste relevant sections from the manuscript (include quotes in quotation marks "like this" to indicate direct quotes from your manuscript), or elaborate on this item by providing additional information not in the ms, or briefly explain why the item is not applicable/relevant for your study

"We used the DHARE platform for TM, which was developed by coauthor Dr Del Fiol and other members of the ReImagine EHR program of the University of Utah Department of Biomedical Informatics. DHARE has been supported by multiple grants from the National Institutes of Health, awarded to investigators from the University of Utah Department of Biomedical Informatics and Huntsman Cancer Institute. DHARE is a scalable, open-source software platform"

5-ii) Describe the history/development process

Describe the history/development process of the application and previous formative evaluations (e.g., focus groups, usability testing), as these will have an impact on adoption/use rates and help with interpreting results.

1 2 3 4 5

subitem not at all important ○ ○ ○ ○ ○ essential

Does your paper address subitem 5-ii?

Copy and paste relevant sections from the manuscript (include quotes in quotation marks "like this" to indicate direct quotes from your manuscript), or elaborate on this item by providing additional information not in the ms, or briefly explain why the item is not applicable/relevant for your study

"The messaging for this trial was based on a prior trial that used DHARE to deliver a COVID-testing text message intervention, with content informed by the most recent state and federal public health guidance as well as a pediatric infectious disease specialist."

5-iii) Revisions and updating

Revisions and updating. Clearly mention the date and/or version number of the application/intervention (and comparator, if applicable) evaluated, or describe whether the intervention underwent major changes during the evaluation process, or whether the development and/or content was "frozen" during the trial. Describe dynamic components such as news feeds or changing content which may have an impact on the replicability of the intervention (for unexpected events see item 3b).

1 2 3 4 5

subitem not at all important ○ ○ ○ ○ ○ essential

Does your paper address subitem 5-iii?

Copy and paste relevant sections from the manuscript (include quotes in quotation marks "like this" to indicate direct quotes from your manuscript), or elaborate on this item by providing additional information not in the ms, or briefly explain why the item is not applicable/relevant for your study

"The messaging for this trial was based on a prior trial that used DHARE to deliver a COVID-testing text message intervention, with content informed by the most recent state and federal public health guidance as well as a pediatric infectious disease specialist. Due to changing COVID-19 testing public health guidance over the course of the study, approximately 2 months into the study, intervention messaging shifted away from COVID-19 testing for diagnostic purposes and toward testing for both diagnostic and preventative purposes."

5-iv) Quality assurance methods

Provide information on quality assurance methods to ensure accuracy and quality of information provided [1], if applicable.

1 2 3 4 5

subitem not at all important ○ ○ ○ ○ ○ essential

Does your paper address subitem 5-iv?

Copy and paste relevant sections from the manuscript (include quotes in quotation marks "like this" to indicate direct quotes from your manuscript), or elaborate on this item by providing additional information not in the ms, or briefly explain why the item is not applicable/relevant for your study

"DHARE has supported the creation and implementation of digital health interventions in 7 pragmatic trials addressing health equity in collaboration with 13 Utah Community Health Center systems and their approximately 40 primary care clinics, and with Medicaid patients served by University of Utah Health. The messaging for this trial was based on a prior trial that used DHARE to deliver a COVID-testing text message intervention, with content informed by the most recent state and federal public health guidance as well as a pediatric infectious disease specialist."

5-v) Ensure replicability by publishing the source code, and/or providing screenshots/screen-capture video, and/or providing flowcharts of the algorithms used

Ensure replicability by publishing the source code, and/or providing screenshots/screen-capture video, and/or providing flowcharts of the algorithms used. Replicability (i.e., other researchers should in principle be able to replicate the study) is a hallmark of scientific reporting.

1 2 3 4 5

subitem not at all important ○ ○ ○ ○ ○ essential

Does your paper address subitem 5-v?

Copy and paste relevant sections from the manuscript (include quotes in quotation marks "like this" to indicate direct quotes from your manuscript), or elaborate on this item by providing additional information not in the ms, or briefly explain why the item is not applicable/relevant for your study

"The messaging cycle of the study's longest running text message campaign (May 2022 to June 2023) is shown in Multimedia Appendix 2."

5-vi) Digital preservation

Digital preservation: Provide the URL of the application, but as the intervention is likely to change or disappear over the course of the years; also make sure the intervention is archived (Internet Archive, webcitation.org, and/or publishing the source code or screenshots/videos alongside the article). As pages behind login screens cannot be archived, consider creating demo pages which are accessible without login.

1 2 3 4 5

subitem not at all important ○ ○ ○ ○ ○ essential

Does your paper address subitem 5-vi?

Copy and paste relevant sections from the manuscript (include quotes in quotation marks "like this" to indicate direct quotes from your manuscript), or elaborate on this item by providing additional information not in the ms, or briefly explain why the item is not applicable/relevant for your study

N/A- assessments were web-based, but the intervention was not.

5-vii) Access

Access: Describe how participants accessed the application, in what setting/context, if they had to pay (or were paid) or not, whether they had to be a member of specific group. If known, describe how participants obtained "access to the platform and Internet" [1]. To ensure access for editors/reviewers/readers, consider to provide a "backdoor" login account or demo mode for reviewers/readers to explore the application (also important for archiving purposes, see vi).

1 2 3 4 5

subitem not at all important ○ ○ ○ ○ ○ essential

Does your paper address subitem 5-vii? *

Copy and paste relevant sections from the manuscript (include quotes in quotation marks "like this" to indicate direct quotes from your manuscript), or elaborate on this item by providing additional information not in the ms, or briefly explain why the item is not applicable/relevant for your study

UC: "received unidirectional, static text messages every 3 weeks that mirrored public health messages about COVID-19 testing"
TM: "sent bidirectional text messages related to COVID-19 testing, where the participant could engage with messaging"; cycles lasted 28-30 days
All parents: "provided access to at-home COVID-19 test kits, free of charge."
"All parents who completed both the assessment phase text messages and the survey in a particular month were entered into that month's raffle for a $100 USD gift card"; "participants in the study could receive compensation for completing other assessments not included in this analysis."

5-viii) Mode of delivery, features/functionalities/components of the intervention and comparator, and the theoretical framework

Describe mode of delivery, features/functionalities/components of the intervention and comparator, and the theoretical framework [6] used to design them (instructional strategy [1], behaviour change techniques, persuasive features, etc., see e.g., [7, 8] for terminology). This includes an in-depth description of the content (including where it is coming from and who developed it) [1]," whether [and how] it is tailored to individual circumstances and allows users to track their progress and receive feedback" [6]. This also includes a description of communication delivery channels and – if computer-mediated communication is a component – whether communication was synchronous or asynchronous [6]. It also includes information on presentation strategies [1], including page design principles, average amount of text on pages, presence of hyperlinks to other resources, etc. [1].

1 2 3 4 5

subitem not at all important ○ ○ ○ ○ ○ essential

Does your paper address subitem 5-viii? *

Copy and paste relevant sections from the manuscript (include quotes in quotation marks "like this" to indicate direct quotes from your manuscript), or elaborate on this item by providing additional information not in the ms, or briefly explain why the item is not applicable/relevant for your study

All parents: "provided access to at-home COVID-19 test kits, free of charge"; informed of test kit availability locations via text message.
"The messaging for this trial was based on a prior trial that used DHARE to deliver a COVID-testing text message intervention, with content informed by the most recent state and federal public health guidance as well as a pediatric infectious disease specialist."
UC: "received unidirectional, static text messages every 3 weeks that mirrored public health messages about COVID-19 testing."
TM: "sent bidirectional text messages related to COVID-19 testing"
TM+HN: "Each cycle could also include 1 telephone call from 1 of 2 health navigators"
For more information see manuscript headings "UC Arm" and "Intervention Arm: TM and HN"

5-ix) Describe use parameters

Describe use parameters (e.g., intended "doses" and optimal timing for use). Clarify what instructions or recommendations were given to the user, e.g., regarding timing, frequency, heaviness of use, if any, or was the intervention used ad libitum.

1 2 3 4 5

subitem not at all important ○ ○ ○ ○ ○ essential

Does your paper address subitem 5-ix?

Copy and paste relevant sections from the manuscript (include quotes in quotation marks "like this" to indicate direct quotes from your manuscript), or elaborate on this item by providing additional information not in the ms, or briefly explain why the item is not applicable/relevant for your study

"The entire text message cycle for all intervention groups was approximately 30 days, with the intervention cycle lasting for 23 of those days and the assessment cycle lasting for 7 of those days."
TM: each cycle 28-30 days, dependent on "changing testing recommendations during the pandemic"; intervention: 21-23 days; assessment: 7 days; "The number of text messages sent to each participant per cycle varied from 3 to 13 and was dependent on the participant's responses, with the intervention consisting of 2-8 text messages...and the assessment consisting of 1-5 text messages."

5-x) Clarify the level of human involvement

Clarify the level of human involvement (care providers or health professionals, also technical assistance) in the e-intervention or as co-intervention (detail number and expertise of professionals involved, if any, as well as "type of assistance offered, the timing and frequency of the support, how it is initiated, and the medium by which the assistance is delivered". It may be necessary to distinguish between the level of human involvement required for the trial, and the level of human involvement required for a routine application outside of a RCT setting (discuss under item 21 – generalizability).

1 2 3 4 5

subitem not at all important ○ ○ ○ ○ ○ essential

Does your paper address subitem 5-x?

Copy and paste relevant sections from the manuscript (include quotes in quotation marks "like this" to indicate direct quotes from your manuscript), or elaborate on this item by providing additional information not in the ms, or briefly explain why the item is not applicable/relevant for your study

"Each cycle of intensive TM+HN could also include 1 telephone call from 1 of 2 health navigators focused on addressing testing barriers and providing information on accessing testing"; those "who had been sent a text message recommending a COVID-19 test but who did not report completing a test were placed in a call queue"; "calls were made...(7:30 AM to 10:30 AM, 10:30 AM to 3:00 PM, and 3:00 PM to 6:00 PM). If a participant did not answer a call, the participant was called the next day during a different time slot. If that call was also missed, a final attempt was made the following day during the last time slot that a navigator had not yet used"; "Navigators completed case management training and were trained in an empirically validated behavior change approach (Motivation and Problem Solving)"; "available in English and Spanish."

5-xi) Report any prompts/reminders used

Report any prompts/reminders used: Clarify if there were prompts (letters, emails, phone calls, SMS) to use the application, what triggered them, frequency etc. It may be necessary to distinguish between the level of prompts/reminders required for the trial, and the level of prompts/reminders for a routine application outside of a RCT setting (discuss under item 21 – generalizability).

1 2 3 4 5

subitem not at all important ○ ○ ○ ○ ○ essential

Does your paper address subitem 5-xi? *

Copy and paste relevant sections from the manuscript (include quotes in quotation marks "like this" to indicate direct quotes from your manuscript), or elaborate on this item by providing additional information not in the ms, or briefly explain why the item is not applicable/relevant for your study

UC: "received unidirectional, static text messages every 3 weeks that mirrored public health messages about COVID-19 testing, such as recommendations to test for COVID-19 disease, offering tests, and testing options through the school and district or via direct mailing through the research project"
TM: "sent bidirectional text messages related to COVID-19 testing, where the participant could engage with messaging. Each "cycle" of TM lasted for 28-30 days"; "intervention lasting for 21-23 days and the assessment lasting for 7 days."; "if the participant did not respond to the first intervention message, it was sent a second time"

5-xii) Describe any co-interventions (incl. training/support)

Describe any co-interventions (incl. training/support): Clearly state any interventions that are provided in addition to the targeted eHealth intervention, as ehealth intervention may not be designed as stand-alone intervention. This includes training sessions and support [1]. It may be necessary to distinguish between the level of training required for the trial, and the level of training for a routine application outside of a RCT setting (discuss under item 21 – generalizability.

1 2 3 4 5

subitem not at all important ○ ○ ○ ○ ○ essential

Does your paper address subitem 5-xii? *

Copy and paste relevant sections from the manuscript (include quotes in quotation marks "like this" to indicate direct quotes from your manuscript), or elaborate on this item by providing additional information not in the ms, or briefly explain why the item is not applicable/relevant for your study

"Each cycle of intensive TM+HN could also include 1 telephone call from 1 of 2 health navigators focused on addressing testing barriers and providing information on accessing testing"

6a) Completely defined pre-specified primary and secondary outcome measures, including how and when they were assessed

Does your paper address CONSORT subitem 6a? *

Copy and paste relevant sections from the manuscript (include quotes in quotation marks "like this" to indicate direct quotes from your manuscript), or elaborate on this item by providing additional information not in the ms, or briefly explain why the item is not applicable/relevant for your study

All intervention groups: cycle "approximately 30 days"; intervention cycle=23 days; assessment cycle=7 days
Primary testing outcome: "whether any household members used a COVID-19 test in the previous 30 days"; "Assessment phase text message cycles collected information on the number of household members tested, test results...and names of children who tested positive during the intervention cycle"
Secondary outcome: "the average number of monthly days missed per participating child"; "number of missed school days for students was collected through web-based REDCap surveys delivered monthly"; "parents were asked to report the number of school days their children missed due to COVID-19 symptoms, exposure, quarantine, and testing."

6a-i) Online questionnaires: describe if they were validated for online use and apply CHERRIES items to describe how the questionnaires were designed/deployed

If outcomes were obtained through online questionnaires, describe if they were validated for online use and apply CHERRIES items to describe how the questionnaires were designed/deployed [9].

1 2 3 4 5

subitem not at all important ○ ○ ○ ○ ○ essential

Does your paper address subitem 6a-i?

Copy and paste relevant sections from manuscript text

"available in both English and Spanish."
Surveys: web-based; deployed through REDCap (secure web platform); parents emailed survey monthly; closed (accessible only via survey link); "branded with the institutional affiliation"; 2-22 non-randomized items (varied "depending on the number of children in the family and whether any family members completed COVID-19 testing" split across multiple pages; "a button allowed survey takers to return to previous pages of the survey to change...answers"; "system records all data, even if the survey taker does not press the submission button…incomplete surveys could be "submitted." Items drawn from RADx-UP Common Data Elements and "not specifically validated for web-based use"

6a-ii) Describe whether and how "use" (including intensity of use/dosage) was defined/measured/monitored

Describe whether and how "use" (including intensity of use/dosage) was defined/measured/monitored (logins, logfile analysis, etc.). Use/adoption metrics are important process outcomes that should be reported in any ehealth trial.

1 2 3 4 5

subitem not at all important ○ ○ ○ ○ ○ essential

Does your paper address subitem 6a-ii?

Copy and paste relevant sections from manuscript text

Due to the in-depth nature of the use data, we are reporting it in a separate publication.

6a-iii) Describe whether, how, and when qualitative feedback from participants was obtained

Describe whether, how, and when qualitative feedback from participants was obtained

Describe whether, how, and when qualitative feedback from participants was obtained (e.g., through emails, feedback forms, interviews, focus groups).

1 2 3 4 5

subitem not at all important ○ ○ ○ ○ ○ essential

Does your paper address subitem 6a-iii?
Copy and paste relevant sections from manuscript text

Qualitative data was requested from participants, but due to the in-depth nature of the data, we are reporting it in a separate publication.

6b) Any changes to trial outcomes after the trial commenced, with reasons

Does your paper address CONSORT subitem 6b? *
Copy and paste relevant sections from the manuscript (include quotes in quotation marks "like this" to indicate direct quotes from your manuscript), or elaborate on this item by providing additional information not in the ms, or briefly explain why the item is not applicable/relevant for your study

"As mentioned in the "Design" section earlier, the summary measure of interest changed during enrollment because the rate of participants qualifying as nonresponders and the overall self-report rate were too low to make meaningful comparisons between TM and TM+HN. The summary measures were estimated, allowing for the TM effect to vary over calendar time."

7a) How sample size was determined
NPT: When applicable, details of whether and how the clustering by care provides or centers was addressed

7a-i) Describe whether and how expected attrition was taken into account when calculating the sample size
Describe whether and how expected attrition was taken into account when calculating the sample size.

1 2 3 4 5

subitem not at all important ○ ○ ○ ○ ○ essential

Does your paper address subitem 7a-i?
Copy and paste relevant sections from manuscript title (include quotes in quotation marks "like this" to indicate direct quotes from your manuscript), or elaborate on this item by providing additional information not in the ms, or briefly explain why the item is not applicable/relevant for your study

"Changes from the original study design and sample size considerations are documented in the statistical analysis plan (Multimedia Appendix 1)."
"Throughout the study, the power to detect small to moderate effect sizes was reassessed while making the most conservative assumptions about missing data. The end sample size of 7122 was determined based upon simulations, which were more than 80.0% powered to detect a treatment effect of 0.30 among self-reported outcomes and conjectured an equal testing rate among unreported outcomes (we considered this a conservative assumption)."

7b) When applicable, explanation of any interim analyses and stopping guidelines

Does your paper address CONSORT subitem 7b? *
Copy and paste relevant sections from the manuscript (include quotes in quotation marks "like this" to indicate direct quotes from your manuscript), or elaborate on this item by providing additional information not in the ms, or briefly explain why the item is not applicable/relevant for your study

This question is not applicable to our study.

8a) Method used to generate the random allocation sequence
NPT: When applicable, how care providers were allocated to each trial group

Does your paper address CONSORT subitem 8a? *
Copy and paste relevant sections from the manuscript (include quotes in quotation marks "like this" to indicate direct quotes from your manuscript), or elaborate on this item by providing additional information not in the ms, or briefly explain why the item is not applicable/relevant for your study

"The random allocation sequence was generated by the study statistician (JC) using R's randomizeR package (R Project for Statistical Computing) and implemented automatically by the in-house Digital Health to Advance Research Equity (DHARE) software...which automatically assigns participants to study arms based on the randomization sequence produced by R."

8b) Type of randomisation; details of any restriction (such as blocking and block size)

Does your paper address CONSORT subitem 8b? *
Copy and paste relevant sections from the manuscript (include quotes in quotation marks "like this" to indicate direct quotes from your manuscript), or elaborate on this item by providing additional information not in the ms, or briefly explain why the item is not applicable/relevant for your study

"participants were randomized...using Soares and Wu's Big Stick Design with a maximum tolerable imbalance of 3 to maintain the desired allocation ratio and to reduce the risk of chronological imbalances between treatment arms over time"

9) Mechanism used to implement the random allocation sequence (such as sequentially numbered containers), describing any steps taken to conceal the sequence until interventions were assigned

Does your paper address CONSORT subitem 9? *
Copy and paste relevant sections from the manuscript (include quotes in quotation marks "like this" to indicate direct quotes from your manuscript), or elaborate on this item by providing additional information not in the ms, or briefly explain why the item is not applicable/relevant for your study

"The random allocation sequence was generated by the study statistician (JC) using R's randomizeR package...and implemented automatically by the in-house Digital Health to Advance Research Equity (DHARE) software...which automatically assigns participants to study arms based on the randomization sequence produced by R. This enabled the allocation to be concealed from investigators and study team."

10) Who generated the random allocation sequence, who enrolled participants, and who assigned participants to interventions

Does your paper address CONSORT subitem 10? *
Copy and paste relevant sections from the manuscript (include quotes in quotation marks "like this" to indicate direct quotes from your manuscript), or elaborate on this item by providing additional information not in the ms, or briefly explain why the item is not applicable/relevant for your study

"The random allocation sequence was generated by the study statistician (JC)"
"All participants were assigned a first- and second-level randomization by the study statistician at the start of the study and were unblinded to the intervention."
Because the parents at each school were automatically enrolled if they didn't opt out, there were no individuals enrolling participants.

11a) If done, who was blinded after assignment to interventions (for example, participants, care providers, those assessing outcomes) and how
NPT: Whether or not administering co-interventions were blinded to group assignment

11a-i) Specify who was blinded, and who wasn't
Specify who was blinded, and who wasn't. Usually, in web-based trials it is not possible to blind the participants [1, 3] (this should be clearly acknowledged), but it may be possible to blind outcome assessors, those doing data analysis or those administering co-interventions (if any).

1 2 3 4 5

subitem not at all important ○ ○ ○ ○ ○ essential

Does your paper address subitem 11a-i? *
Copy and paste relevant sections from the manuscript (include quotes in quotation marks "like this" to indicate direct quotes from your manuscript), or elaborate on this item by providing additional information not in the ms, or briefly explain why the item is not applicable/relevant for your study

"All participants...were unblinded to the intervention."
"The outcome assessors and data analysts were not blinded to which intervention was the intervention of interest."

11a-ii) Discuss e.g., whether participants knew which intervention was the "intervention of interest" and which one was the "comparator"
Informed consent procedures (4a-ii) can create biases and certain expectations - discuss e.g., whether participants knew which intervention was the "intervention of interest" and which one was the "comparator".

1 2 3 4 5

subitem not at all important ○ ○ ○ ○ ○ essential

Does your paper address subitem 11a-ii?
Copy and paste relevant sections from the manuscript (include quotes in quotation marks "like this" to indicate direct quotes from your manuscript), or elaborate on this item by providing additional information not in the ms, or briefly explain why the item is not applicable/relevant for your study

The online consent form provided a description of all interventions without labeling which was the control intervention and which was the intervention of interest.

11b) If relevant, description of the similarity of interventions
(this item is usually not relevant for ehealth trials as it refers to similarity of a placebo or sham intervention to a active medication/intervention)

Does your paper address CONSORT subitem 11b? *
Copy and paste relevant sections from the manuscript (include quotes in quotation marks "like this" to indicate direct quotes from your manuscript), or elaborate on this item by providing additional information not in the ms, or briefly explain why the item is not applicable/relevant for your study

Each intervention used text messaging, but one had additional features (bidirectional messaging, health navigation).

12a) Statistical methods used to compare groups for primary and secondary outcomes
NPT: When applicable, details of whether and how the clustering by care providers or centers was addressed

Does your paper address CONSORT subitem 12a? *
Copy and paste relevant sections from the manuscript (include quotes in quotation marks "like this" to indicate direct quotes from your manuscript), or elaborate on this item by providing additional information not in the ms, or briefly explain why the item is not applicable/relevant for your study

"RTR was estimated separately for each era of allocation enrollment...and then aggregated as a meta-analysis"; "For each allocation era, we used generalized estimating equations with a logit link to estimate the treatment-specific rate of testing over follow-up calendar time. We clustered by participant and used an exchangeable working model with robust standard errors. The model included treatment, calendar date modeled with restricted cubic splines having 5 knots, and their interaction." For further information see manuscript heading "Analytic Plan," paragraph 2.
"RMD 30, was estimated following a similar strategy as the RTR; however, we used a Gaussian generalized estimating equations link rather than a logit link"; "used only self-reported outcomes that occurred when school was in session between August 2022 and May 2023" which excluded one school

12a-i) Imputation techniques to deal with attrition / missing values
Imputation techniques to deal with attrition / missing values: Not all participants will use the intervention/comparator as intended and attrition is typically high in ehealth trials. Specify how participants who did not use the application or dropped out from the trial were treated in the statistical analysis (a complete case analysis is strongly discouraged, and simple imputation techniques such as LOCF may also be problematic [4]).

1 2 3 4 5

subitem not at all important ○ ○ ○ ○ ○ essential

Does your paper address subitem 12a-i? *
Copy and paste relevant sections from the manuscript (include quotes in quotation marks "like this" to indicate direct quotes from your manuscript), or elaborate on this item by providing additional information not in the ms, or briefly explain why the item is not applicable/relevant for your study

"For analyses that used SVI or the number of enrolled children, missing data were imputed using mean imputation conditioned on school enrollment"
RTR: "The first sensitivity analysis used a pattern-mixture model with the assumption that outcomes were predictable from observed baseline and nonbaseline covariates (ie, MAR)"
RMD 30: "We performed a similar suite of sensitivity analyses using the pattern-mixture model and tipping point analyses"

12b) Methods for additional analyses, such as subgroup analyses and adjusted analyses

Does your paper address CONSORT subitem 12b? *
Copy and paste relevant sections from the manuscript (include quotes in quotation marks "like this" to indicate direct quotes from your manuscript), or elaborate on this item by providing additional information not in the ms, or briefly explain why the item is not applicable/relevant for your study

RTR: "The first sensitivity analysis used a pattern-mixture model with the assumption that outcomes were predictable from observed baseline and nonbaseline covariates (ie, MAR)"; "We also performed tipping point analyses that conjectured (ie, not assuming MAR) what the RTR would need to be to nullify results"
RMD 30: "We performed a similar suite of sensitivity analyses using the pattern-mixture model and tipping point analyses."
For further information on sensitivity analyses, see manuscript heading "Analytic Plan," paragraphs 3 and 4.
"For both RTR and RMD 30, we tested for an SVI effect modification. Following a similar strategy described for RTR and RMD 30, we estimated and tested for any added (or diminished) treatment effect when receiving TM between subgroups"; "We assessed for effect modification under observed outcomes and the pattern-mixture model."

X26) REB/IRB Approval and Ethical Considerations [recommended as subheading under "Methods"] (not a CONSORT item)

X26-i) Comment on ethics committee approval

1 2 3 4 5

subitem not at all important ○ ○ ○ ○ ○ essential

Does your paper address subitem X26-i?
Copy and paste relevant sections from the manuscript (include quotes in quotation marks "like this" to indicate direct quotes from your manuscript), or elaborate on this item by providing additional information not in the ms, or briefly explain why the item is not applicable/relevant for your study

"This study was approved by the University of Utah IRB (IRB_00143340). The study received a waiver of documentation of consent from the IRB, allowing for expedited recruitment and enrollment."

x26-ii) Outline informed consent procedures
Outline informed consent procedures e.g., if consent was obtained offline or online (how? Checkbox, etc.?), and what information was provided (see 4a-ii). See [6] for some items to be included in informed consent documents.

1 2 3 4 5

subitem not at all important ○ ○ ○ ○ ○ essential

Does your paper address subitem X26-ii?
Copy and paste relevant sections from the manuscript (include quotes in quotation marks "like this" to indicate direct quotes from your manuscript), or elaborate on this item by providing additional information not in the ms, or briefly explain why the item is not applicable/relevant for your study

"The study received a waiver of documentation of consent from the IRB, allowing for expedited recruitment and enrollment."
"parents were sent an initial Health Insurance Portability and Accountability Act–compliant bidirectional text message that described the study and stated its affiliation with the University of Utah, provided a link to the study website and consent form...and the opportunity to opt out of the program. The consent form on the study website was text-based but brief (approximately 300 words), written at a Flesch-Kincaid Grade Level of 7.4, and available in both English and Spanish."

X26-iii) Safety and security procedures
Safety and security procedures, incl. privacy considerations, and any steps taken to reduce the likelihood or detection of harm (e.g., education and training, availability of a hotline)

1 2 3 4 5

subitem not at all important ○ ○ ○ ○ ○ essential

Does your paper address subitem X26-iii?
Copy and paste relevant sections from the manuscript (include quotes in quotation marks "like this" to indicate direct quotes from your manuscript), or elaborate on this item by providing additional information not in the ms, or briefly explain why the item is not applicable/relevant for your study

"All participants were given the contact information of the research team and were able to contact them by text, phone, or email with any safety or security concerns. The privacy and confidentiality of all participants' data were maintained using REDCap, a secure web-based platform for building, dispensing, and collecting information from surveys. All data were anonymized prior to analysis."

RESULTS

13a) For each group, the numbers of participants who were randomly assigned, received intended treatment, and were analysed for the primary outcome
NPT: The number of care providers or centers performing the intervention in each group and the number of patients treated by each care provider in each center

Does your paper address CONSORT subitem 13a? *
Copy and paste relevant sections from the manuscript (include quotes in quotation marks "like this" to indicate direct quotes from your manuscript), or elaborate on this item by providing additional information not in the ms, or briefly explain why the item is not applicable/relevant for your study

See CONSORT flow diagram (Figure 2)

13b) For each group, losses and exclusions after randomisation, together with reasons

Does your paper address CONSORT subitem 13b? (NOTE: Preferably, this is shown in a CONSORT flow diagram) *
Copy and paste relevant sections from the manuscript (include quotes in quotation marks "like this" to indicate direct quotes from your manuscript), or elaborate on this item by providing additional information not in the ms, or briefly explain why the item is not applicable/relevant for your study

See CONSORT flow diagram (Figure 2)

13b-i) Attrition diagram
Strongly recommended: An attrition diagram (e.g., proportion of participants still logging in or using the intervention/comparator in each group plotted over time, similar to a survival curve) or other figures or tables demonstrating usage/dose/engagement.

1 2 3 4 5

subitem not at all important ○ ○ ○ ○ ○ essential

Does your paper address subitem 13b-i?
Copy and paste relevant sections from the manuscript or cite the figure number if applicable (include quotes in quotation marks "like this" to indicate direct quotes from your manuscript), or elaborate on this item by providing additional information not in the ms, or briefly explain why the item is not applicable/relevant for your study

Due to the in-depth nature of the use data, we are reporting on it in a separate paper.

14a) Dates defining the periods of recruitment and follow-up

Does your paper address CONSORT subitem 14a? *
Copy and paste relevant sections from the manuscript (include quotes in quotation marks "like this" to indicate direct quotes from your manuscript), or elaborate on this item by providing additional information not in the ms, or briefly explain why the item is not applicable/relevant for your study

"The study recruited participants between February 2022 and May 2023, began follow-up of outcomes in March 2022, and ended follow-up in June 2023."

14a-i) Indicate if critical "secular events" fell into the study period
Indicate if critical "secular events" fell into the study period, e.g., significant changes in Internet resources available or "changes in computer hardware or Internet delivery resources"

1 2 3 4 5

subitem not at all important ○ ○ ○ ○ ○ essential

Does your paper address subitem 14a-i?
Copy and paste relevant sections from the manuscript (include quotes in quotation marks "like this" to indicate direct quotes from your manuscript), or elaborate on this item by providing additional information not in the ms, or briefly explain why the item is not applicable/relevant for your study

Not applicable- no secular events.

14b) Why the trial ended or was stopped (early)

Does your paper address CONSORT subitem 14b? *
Copy and paste relevant sections from the manuscript (include quotes in quotation marks "like this" to indicate direct quotes from your manuscript), or elaborate on this item by providing additional information not in the ms, or briefly explain why the item is not applicable/relevant for your study

"This study concluded when the 2022-2023 school year ended, near the end of the project's funding period"

15) A table showing baseline demographic and clinical characteristics for each group
NPT: When applicable, a description of care providers (case volume, qualification, expertise, etc.) and centers (volume) in each group

Does your paper address CONSORT subitem 15? *
Copy and paste relevant sections from the manuscript (include quotes in quotation marks "like this" to indicate direct quotes from your manuscript), or elaborate on this item by providing additional information not in the ms, or briefly explain why the item is not applicable/relevant for your study

See Table 1 for this information

15-i) Report demographics associated with digital divide issues
In ehealth trials it is particularly important to report demographics associated with digital divide issues, such as age, education, gender, social-economic status, computer/Internet/ehealth literacy of the participants, if known.

1 2 3 4 5

subitem not at all important ○ ○ ○ ○ ○ essential

Does your paper address subitem 15-i? *
Copy and paste relevant sections from the manuscript (include quotes in quotation marks "like this" to indicate direct quotes from your manuscript), or elaborate on this item by providing additional information not in the ms, or briefly explain why the item is not applicable/relevant for your study

"Half (50.0%; 8/16) of participating schools were title 1 schools...less than half (39.9%; 3840/9633) of the student body was eligible for free or reduced lunch"; "SVI for students across all 16 schools had a range of 0.0014-1, with a mean score of 0.58 (median 0.58)"; "Table 1 shows demographic characteristics of students from the largest participating school district (14 schools) based on data provided by the school district"; "Most of the participating parents were female (75.0%; 5340/7122)."

16) For each group, number of participants (denominator) included in each analysis and whether the analysis was by original assigned groups

16-i) Report multiple "denominators" and provide definitions
Report multiple "denominators" and provide definitions: Report N's (and effect sizes) "across a range of study participation [and use] thresholds" [1], e.g., N exposed, N consented, N used more than x times, N used more than y weeks, N participants "used" the intervention/comparator at specific pre-defined time points of interest (in absolute and relative numbers per group). Always clearly define "use" of the intervention.

1 2 3 4 5

subitem not at all important ○ ○ ○ ○ ○ essential

Does your paper address subitem 16-i? *
Copy and paste relevant sections from the manuscript (include quotes in quotation marks "like this" to indicate direct quotes from your manuscript), or elaborate on this item by providing additional information not in the ms, or briefly explain why the item is not applicable/relevant for your study

See Figure 2. Due to the in-depth nature of the use data, we are reporting on it in a separate manuscript.

16-ii) Primary analysis should be intent-to-treat
Primary analysis should be intent-to-treat, secondary analyses could include comparing only "users", with the appropriate caveats that this is no longer a randomized sample (see 18-i).

1 2 3 4 5

subitem not at all important ○ ○ ○ ○ ○ essential

Does your paper address subitem 16-ii?
Copy and paste relevant sections from the manuscript (include quotes in quotation marks "like this" to indicate direct quotes from your manuscript), or elaborate on this item by providing additional information not in the ms, or briefly explain why the item is not applicable/relevant for your study

In our SAP on clinicaltrials.gov, we specified the complete case analysis as the primary analysis with extensive sensitivity analyses. Under the missing at random (MAR) assumption, a complete outcomes analysis is approximately unbiased and is often used as a starting point analysis. However, we fully agree that this does not hold to the ITT principle that uses outcomes from all randomized participants. The sensitivity analyses are more model dependent yet hold to the ITT principle. They include (1) a pattern mixture model which makes a MAR assumption, (2) conjectures of the missing outcome responses which makes a missing not at random assumption, and (3) tipping point analyses to indicate the modifications from the pattern mixture model that would nullify trial results.

17a) For each primary and secondary outcome, results for each group, and the estimated effect size and its precision (such as 95% confidence interval)

Does your paper address CONSORT subitem 17a? *
Copy and paste relevant sections from the manuscript (include quotes in quotation marks "like this" to indicate direct quotes from your manuscript), or elaborate on this item by providing additional information not in the ms, or briefly explain why the item is not applicable/relevant for your study

"Aggregating across allocation eras, the testing rate was 1.5 times higher in the TM arm than in the UC arm (21.6% vs 14.4%, RTR = 1.50, 95% CI 1.35-1.67; P<.001; Table 2 and Figure 3A)."
"Aggregating across allocation eras, the analysis showed no significant difference in the number of school days between UC and TM; there was insufficient evidence to observe a difference in the 30-day missed school rate (0.43 per month vs 0.28 in UC, RMD 30 = 1.55, 95% CI 0.98-2.45; P=.06; Table 4 and Figure 4A)."

17a-i) Presentation of process outcomes such as metrics of use and intensity of use
In addition to primary/secondary (clinical) outcomes, the presentation of process outcomes such as metrics of use and intensity of use (dose, exposure) and their operational definitions is critical. This does not only refer to metrics of attrition (13-b) (often a binary variable), but also to more continuous exposure metrics such as "average session length". These must be accompanied by a technical description how a metric like a "session" is defined (e.g., timeout after idle time) [1] (report under item 6a).

1 2 3 4 5

subitem not at all important ○ ○ ○ ○ ○ essential

Does your paper address subitem 17a-i?
Copy and paste relevant sections from the manuscript (include quotes in quotation marks "like this" to indicate direct quotes from your manuscript), or elaborate on this item by providing additional information not in the ms, or briefly explain why the item is not applicable/relevant for your study

"low self-report rate of outcomes (35.3% (2517/7122) of participants self-reported on testing at least once, 19.8% (9698/48878) of all possible testing outcomes were self-reported, 9.1% (642/7021) of participants self-reported missed school days, and 6.0% (2553/42538) of all missed school day outcomes were self-reported)."

"On average, participants received 7 opportunities to self-report (IQR 2-10 opportunities)." The number of people who responded to the messages is outside the scope of this paper; in a separate manuscript, we are reporting how many people responded to the messages at all stages of the study.

17b) For binary outcomes, presentation of both absolute and relative effect sizes is recommended

Does your paper address CONSORT subitem 17b? *

Copy and paste relevant sections from the manuscript (include quotes in quotation marks "like this" to indicate direct quotes from your manuscript), or elaborate on this item by providing additional information not in the ms, or briefly explain why the item is not applicable/relevant for your study

We have provided the relative testing rate (RTR) for the binary testing outcome, and an estimate of the testing rate for each arm.

18) Results of any other analyses performed, including subgroup analyses and adjusted analyses, distinguishing pre-specified from exploratory

Does your paper address CONSORT subitem 18? *

Copy and paste relevant sections from the manuscript (include quotes in quotation marks "like this" to indicate direct quotes from your manuscript), or elaborate on this item by providing additional information not in the ms, or briefly explain why the item is not applicable/relevant for your study

"Under the pattern-mixture model sensitivity analysis, the testing rate aggregated across allocation eras was 22.8% in the TM arm versus 13.5% in the UC arm"; "the RTR still favored the TM arm" regardless of how unreported testing was interpreted; "We did not observe differential effects by SVI subgroups"
"Under the pattern-mixture model sensitivity analysis, the effect of the TM upon relative missed days rate showed a trend but was even less pronounced"; "Figure 4B provides scenarios for which the unreported school days among UC and TM would be enough to observe a TM effect"
"did not observe differential effects by SVI subgroups" for either testing rate or missed days rate.

18-i) Subgroup analysis of comparing only users

A subgroup analysis of comparing only users is not uncommon in ehealth trials, but if done, it must be stressed that this is a self-selected sample and no longer an unbiased sample from a randomized trial (see 16-iii).

1 2 3 4 5

subitem not at all important ○ ○ ○ ○ ○ essential

Does your paper address subitem 18-i?

Copy and paste relevant sections from the manuscript (include quotes in quotation marks "like this" to indicate direct quotes from your manuscript), or elaborate on this item by providing additional information not in the ms, or briefly explain why the item is not applicable/relevant for your study

Your answer

19) All important harms or unintended effects in each group
(for specific guidance see CONSORT for harms)

Does your paper address CONSORT subitem 19? *

Copy and paste relevant sections from the manuscript (include quotes in quotation marks "like this" to indicate direct quotes from your manuscript), or elaborate on this item by providing additional information not in the ms, or briefly explain why the item is not applicable/relevant for your study

"The interventions had no noted harms or unintended effects, including any that could have been reported to the IRB."

19-i) Include privacy breaches, technical problems

Include privacy breaches, technical problems. This does not only include physical "harm" to participants, but also incidents such as perceived or real privacy breaches [1], technical problems, and other unexpected/unintended incidents. "Unintended effects" also includes unintended positive effects [2].

1 2 3 4 5

subitem not at all important ○ ○ ○ ○ ○ essential

Does your paper address subitem 19-i?

Copy and paste relevant sections from the manuscript (include quotes in quotation marks "like this" to indicate direct quotes from your manuscript), or elaborate on this item by providing additional information not in the ms, or briefly explain why the item is not applicable/relevant for your study

"DHARE randomized 61 participants in the UC arm and 119 participants in the TM arm a second time and sent a second initial text message due to a technical error (eg, being listed more than once in the database); we excluded these individuals from analysis"

19-ii) Include qualitative feedback from participants or observations from staff/researchers

Include qualitative feedback from participants or observations from staff/researchers, if available, on strengths and shortcomings of the application, especially if they point to unintended/unexpected effects or uses. This includes (if available) reasons for why people did or did not use the application as intended by the developers.

1 2 3 4 5

subitem not at all important ○ ○ ○ ○ ○ essential

Does your paper address subitem 19-ii?

Copy and paste relevant sections from the manuscript (include quotes in quotation marks "like this" to indicate direct quotes from your manuscript), or elaborate on this item by providing additional information not in the ms, or briefly explain why the item is not applicable/relevant for your study

We have in-depth data from parents/guardians and school staff/administration and due to the in-depth nature of the data, we are publishing it separately from this paper.

DISCUSSION

22) Interpretation consistent with results, balancing benefits and harms, and considering other relevant evidence

NPT: In addition, take into account the choice of the comparator, lack of or partial blinding, and unequal expertise of care providers or centers in each group

22-i) Restate study questions and summarize the answers suggested by the data, starting with primary outcomes and process outcomes (use)

Restate study questions and summarize the answers suggested by the data, starting with primary outcomes and process outcomes (use).

1 2 3 4 5

subitem not at all important ○ ○ ○ ○ ○ essential

Does your paper address subitem 22-i? *

Copy and paste relevant sections from the manuscript (include quotes in quotation marks "like this" to indicate direct quotes from your manuscript), or elaborate on this item by providing additional information not in the ms, or briefly explain why the item is not applicable/relevant for your study

"results indicated that a bidirectional TM intervention...led to increased self-reported testing compared with fewer noninteractive (ie, 1-way) text messages related to COVID-19 testing." "This study did not observe a difference in missed school days between the study arms, suggesting that access to testing did not negatively impact school attendance, although missed school days tended to be higher in the bidirectional texting arm."

22-ii) Highlight unanswered new questions, suggest future research

Highlight unanswered new questions, suggest future research.

1 2 3 4 5

subitem not at all important ○ ○ ○ ○ ○ essential

Does your paper address subitem 22-ii?

Copy and paste relevant sections from the manuscript (include quotes in quotation marks "like this" to indicate direct quotes from your manuscript), or elaborate on this item by providing additional information not in the ms, or briefly explain why the item is not applicable/relevant for your study

Your answer

20) Trial limitations, addressing sources of potential bias, imprecision, and, if relevant, multiplicity of analyses

20-i) Typical limitations in ehealth trials

Typical limitations in ehealth trials: Participants in ehealth trials are rarely blinded. Ehealth trials often look at a multiplicity of outcomes, increasing risk for a Type I error. Discuss biases due to non-use of the intervention/usability issues, biases through informed consent procedures, unexpected events.

1 2 3 4 5

subitem not at all important ○ ○ ○ ○ ○ essential

Does your paper address subitem 20-i? *

Copy and paste relevant sections from the manuscript (include quotes in quotation marks "like this" to indicate direct quotes from your manuscript), or elaborate on this item by providing additional information not in the ms, or briefly explain why the item is not applicable/relevant for your study

"challenges of capturing and maintaining research engagement in a community sample, including through individuals opting out of the study initially or over time and missingness of longitudinal research"; "Those who provided data on self-reported outcomes may represent a self-selected sample"; "results of this study appear to be robust to various assumptions regarding missingness, as seen in the sensitivity analyses. To facilitate participation and engagement of individuals who are not fluent in English or Spanish, consent forms and website materials for future studies could use simpler English, include graphics to aid understanding, and be offered in additional languages. Other limitations include the reliance on self-reported data and focus on a single geographic area, which limits its generalizability."

21) Generalisability (external validity, applicability) of the trial findings

NPT: External validity of the trial findings according to the intervention, comparators, patients, and care providers or centers involved in the trial

21-i) Generalizability to other populations

Generalizability to other populations: In particular, discuss generalizability to a general Internet population, outside of a RCT setting, and general patient population, including applicability of the study results for other organizations

1 2 3 4 5

subitem not at all important ○ ○ ○ ○ ○ essential

Does your paper address subitem 21-i?

Copy and paste relevant sections from the manuscript (include quotes in quotation marks "like this" to indicate direct quotes from your manuscript), or elaborate on this item by providing additional information not in the ms, or briefly explain why the item is not applicable/relevant for your study

"focus on a single geographic area, which limits its generalizability."

21-ii) Discuss if there were elements in the RCT that would be different in a routine application setting

Discuss if there were elements in the RCT that would be different in a routine application setting (e.g., prompts/reminders, more human involvement, training sessions or other co-interventions) and what impact the omission of these elements could have on use, adoption, or outcomes if the intervention is applied outside of a RCT setting.

1 2 3 4 5

subitem not at all important ○ ○ ○ ○ ○ essential

Does your paper address subitem 21-ii?

Copy and paste relevant sections from the manuscript (include quotes in quotation marks "like this" to indicate direct quotes from your manuscript), or elaborate on this item by providing additional information not in the ms, or briefly explain why the item is not applicable/relevant for your study

"the implementation of the intervention and trial procedures involved software programmers who could set up separate study arms and modify the content of the text messages based on the phase of the pandemic and the latest COVID-19 guidance. Outside of this trial setting...school systems could use preexisting TM systems and ideally modify the text messages based on current public health guidance. The intervention could also be implemented using TM systems available on the market, via the platform used in the current trial which is open source, and could be modified if other systems were available...could use...email, social media, and surveys, that included embedded links for additional information."

OTHER INFORMATION

23) Registration number and name of trial registry

Does your paper address CONSORT subitem 23? *

Copy and paste relevant sections from the manuscript (include quotes in quotation marks "like this" to indicate direct quotes from your manuscript), or elaborate on this item by providing additional information not in the ms, or briefly explain why the item is not applicable/relevant for your study

"Trial Registration: ClinicalTrials.gov NCT05112900; http://clinicaltrials.gov/ct2/show/NCT05112900."
"The statistical analysis plan and corresponding analysis, along with the full trial protocol, are detailed at ClinicalTrials.gov (NCT05112900)."

24) Where the full trial protocol can be accessed, if available

Does your paper address CONSORT subitem 24? *

Cite a Multimedia Appendix, other reference, or copy and paste relevant sections from the manuscript (include quotes in quotation marks "like this" to indicate direct quotes from your manuscript), or elaborate on this item by providing additional information not in the ms, or briefly explain why the item is not applicable/relevant for your study

"The statistical analysis plan and corresponding analysis, along with the full trial protocol, are detailed at ClinicalTrials.gov (NCT05112900)."

25) Sources of funding and other support (such as supply of drugs), role of funders

Does your paper address CONSORT subitem 25? *

Copy and paste relevant sections from the manuscript (include quotes in quotation marks "like this" to indicate direct quotes from your manuscript), or elaborate on this item by providing additional information not in the ms, or briefly explain why the item is not applicable/relevant for your study

Please see Aknowledgments section for full funding and aknowledgments, as it is too long to i

X27) Conflicts of Interest (not a CONSORT item)

X27-i) State the relation of the study team towards the system being evaluated

In addition to the usual declaration of interests (financial or otherwise), also state the relation of the study team towards the system being evaluated, i.e., state if the authors/evaluators are distinct from or identical with the developers/sponsors of the intervention.

1 2 3 4 5

subitem not at all important ○ ○ ○ ○ ○ essential

Does your paper address subitem X27-i?

Copy and paste relevant sections from the manuscript (include quotes in quotation marks "like this" to indicate direct quotes from your manuscript), or elaborate on this item by providing additional information not in the ms, or briefly explain why the item is not applicable/relevant for your study

In Methods: "We used the DHARE platform for TM, which was developed by coauthor Dr Del Fiol and other members of the ReImagine EHR program of the University of Utah Department of Biomedical Informatics." We also note that "DHARE is...open-source"

About the CONSORT EHEALTH checklist

As a result of using this checklist, did you make changes in your manuscript? *

○ yes, major changes
◉ yes, minor changes
○ no

What were the most important changes you made as a result of using this checklist?

Added that the full trial protocol could be found on ClinicalTrials.gov

How much time did you spend on going through the checklist INCLUDING making changes in your manuscript *

5 hours- we had completed the checklist prior to our initial submission, so just needed to provide the updated text.

As a result of using this checklist, do you think your manuscript has improved? *

○ yes
○ no
◉ Other: I believe it greatly improved our initial submission, but the second tim

Would you like to become involved in the CONSORT EHEALTH group?

This would involve for example becoming involved in participating in a workshop and writing an "Explanation and Elaboration" document

○ yes
◉ no
○ Other:

Clear selection

Any other comments or questions on CONSORT EHEALTH

I can't tell which responses are too large- it would be helpful to notify which ones are too large, or specify a character limit on the form.

STOP - Save this form as PDF before you click submit

To generate a record that you filled in this form, we recommend to generate a PDF of this page (on a Mac, simply select "print" and then select "print as PDF") before you submit it.

When you submit your (revised) paper to JMIR, please upload the PDF as supplementary file.

Don't worry if some text in the textboxes is cut off, as we still have the complete information in our database. Thank you!

Final step: Click submit !

Click submit so we have your answers in our database!

Submit Clear form

Never submit passwords through Google Forms.

This content is neither created nor endorsed by Google. - Contact form owner - Terms of Service - Privacy Policy

Does this form look suspicious? Report

Google Forms
